# miR-181a/b-1 controls thymic selection of Treg cells and tunes their suppressive capacity

**DOI:** 10.1371/journal.pbio.2006716

**Published:** 2019-03-11

**Authors:** Marcin Łyszkiewicz, Samantha J. Winter, Katrin Witzlau, Lisa Föhse, Rebecca Brownlie, Jacek Puchałka, Nikita A. Verheyden, Heike Kunze-Schumacher, Esther Imelmann, Jonas Blume, Solaiman Raha, Takashi Sekiya, Akihiko Yoshimura, Jochen T. Frueh, Evelyn Ullrich, Jochen Huehn, Siegfried Weiss, Maximiliano G. Gutierrez, Immo Prinz, Rose Zamoyska, Natalia Ziętara, Andreas Krueger

**Affiliations:** 1 Institute of Immunology, Hannover Medical School, Hannover, Germany; 2 Institute for Immunology, Ludwig-Maximilians University, Planegg-Martiensried, Germany; 3 Institute for Molecular Medicine, Goethe University Frankfurt am Main, Frankfurt, Germany; 4 Institute for Immunology and Infection Research, The University of Edinburgh, Edinburgh, United Kingdom; 5 Department of Pediatrics, Dr. von Hauner Kinderspital, Ludwig-Maximilians University, Munich, Germany; 6 Department of Microbiology and Immunology, Keio University School of Medicine, Tokyo, Japan; 7 Experimental Immunology, Department for Children and Adolescents Medicine, Hospital of the Goethe University Frankfurt, Frankfurt, Germany; 8 LOEWE Center for Cell and Gene Therapy, Goethe University, Frankfurt, Germany; 9 Department of Experimental Immunology, Helmholtz Centre for Infection Research, Braunschweig, Germany; 10 The Francis Crick Institute, London, England, United Kingdom; National Cancer Institute, United States of America

## Abstract

The interdependence of selective cues during development of regulatory T cells (Treg cells) in the thymus and their suppressive function remains incompletely understood. Here, we analyzed this interdependence by taking advantage of highly dynamic changes in expression of microRNA 181 family members miR-181a-1 and miR-181b-1 (miR-181a/b-1) during late T-cell development with very high levels of expression during thymocyte selection, followed by massive down-regulation in the periphery. Loss of miR-181a/b-1 resulted in inefficient de novo generation of Treg cells in the thymus but simultaneously permitted homeostatic expansion in the periphery in the absence of competition. Modulation of T-cell receptor (TCR) signal strength in vivo indicated that miR-181a/b-1 controlled Treg-cell formation via establishing adequate signaling thresholds. Unexpectedly, miR-181a/b-1–deficient Treg cells displayed elevated suppressive capacity in vivo, in line with elevated levels of cytotoxic T-lymphocyte–associated 4 (CTLA-4) protein, but not mRNA, in thymic and peripheral Treg cells. Therefore, we propose that intrathymic miR-181a/b-1 controls development of Treg cells and imposes a developmental legacy on their peripheral function.

## Author summary

T cells are pivotal in orchestrating an adaptive immune response. They are produced in the thymus and undergo selection processes resulting in elimination of nonfunctional and self-reactive cells in order to prevent autoimmune disease. One type of T cells, called regulatory T cells (Treg cells), is generated either in the thymus or in the periphery through a process termed agonist selection. Peripheral Treg cells are crucial for the suppression of unwanted immune responses. However, too much suppressive function of Treg cells can also impair immune responses directed against tumors. Here, we have analyzed the mechanisms that regulate this process and show that a microRNA, miR-181a/b-1, controls de novo generation of Treg cells by modulating agonist selection. We show that thymic and peripheral Treg cells deficient in miR-181a/b-1 contain higher levels of the key effector molecule CTLA-4, and as a consequence, such Treg cells display increased suppressive capacity. We observe that the expression of miR-181a/b-1 in peripheral Treg cells is much lower when compared to thymocytes; thus, our study implies that peripheral Treg-cell effector function is imprinted during intrathymic differentiation.

## Introduction

Regulatory T cells (Treg cells) expressing the lineage-defining transcription factor forkhead box protein P3 (Foxp3) form an integral part of the adaptive immune system and function to prevent unwanted immune responses [1,2]. Treg cells are generated during T-cell development in the thymus (thymic [t]Treg cells) as well as via peripheral induction of naive T cells (induced [i]Treg cells). Development of tTreg cells depends on strong T-cell receptor (TCR) signals [3]. Accordingly, tTreg cells are generated when a TCR of a developing T cell recognizes a self-antigen with high affinity, as has been demonstrated in mouse models transgenic for both a TCR and its cognate antigen [4,5] and by analysis of polyclonal superantigen-reactive T cells [6,7]. tTreg cells can develop through two distinct precursor (prec) stages. Some Treg-cell precursors are found within a cluster of differentiation (CD) 4 single-positive (SP) Foxp3^−^, glucocorticoid-induced tumor necrosis factor receptor-related protein high (GITR^hi^), CD25^+^ population [8]. These cells are the first precursors generated in double transgenic TCR/cognate-antigen mouse models [9,10]. More recently, an additional CD4SP Foxp3^+^CD25^−^ Treg-cell precursor has been described [11]. These cells are phenotypically less mature than tTreg cells, are generated with similar kinetics as tTreg cells upon induction of T-cell development in vivo, and efficiently become tTreg cells in vitro and in vivo [10,11]. Generation of both precursors is dependent on strong TCR signals, although on average, Foxp3^+^CD25^–^ Treg-cell precursors have received somewhat weaker signals than their Foxp3^−^GITR^hi^CD25^+^ counterparts [10]. Further differentiation into mature Foxp3^+^CD25^+^ tTreg cells is then dependent on γc cytokines [8,10–12]. The level of TCR signal strength required for tTreg cell generation in comparison to TCR signals resulting in clonal deletion have not been fully established. Data from a TCR signaling reporter as well as repertoire studies suggest that signal strength required for tTreg-cell development overlaps with that inducing clonal deletion in other autoreactive thymocytes [3,13–15]. However, reduction of major histocompatibility complex (MHC) ligand levels on medullary thymic epithelial cells rescued autoreactive T cells from clonal deletion but resulted in a concomitant increase in Treg-cell development, suggesting that at least some tTreg cells are generated through weaker TCR signals than those inducing clonal deletion [16].

Treg cells suppress T-cell immune responses using multiple molecular mechanisms, including consumption of interleukin-2 (IL-2) and production of suppressive cytokines, as well as expression of the coinhibitory receptor cytotoxic T-lymphocyte–associated protein 4 (CTLA-4) [17,18]. Mice specifically lacking the inhibitory receptor CTLA-4 in Treg cells succumb to fatal autoimmune disease, indicating that CTLA-4 plays a major role in suppressive function [19]. It has been proposed that CTLA-4 on Treg cell surfaces acts through capture of the costimulatory ligands CD80 and CD86 on antigen-presenting cells, thereby curtailing full activation of conventional T cells [20].

MicroRNAs (miRNAs) play a critical role in immune homeostasis and tolerance. Global loss of miRNAs results in defective development of tTreg cells [21]. However, no individual miRNA has been demonstrated to control intrathymic generation of Treg cells. miRNA miR-181a is the most prominently expressed miRNA in double-positive (DP) thymocytes, and it has been shown in vitro that miR-181a serves as a rheostat for TCR signals in T cells and thymocytes through targeting a combination of tyrosine and dual-specificity phosphatases, including protein tyrosine phosphatase, nonreceptor type (*Ptpn*) *11*, *Ptpn22*, and dual specificity phosphatase 6 (*Dusp6*) [22–24]. Deletion of miR-181a/b-1 in mice resulted in an almost complete failure in development of invariant natural killer T (iNKT) cells and Mucosal-Associated Invariant T (MAIT) cells [25–27] due to a defect in thymic agonist selection [26,28]. Furthermore, loss of miR-181a/b-1 caused altered selection of conventional T cells in a TCR transgenic model with a shift towards positive selection [29]. However, counterintuitively, miR-181a/b-1^−/−^ mice display increased resistance to experimental autoimmunity, which has not been fully explained [29].

Here, we tested the hypothesis that miR-181a/b-1 controlled intrathymic development of Treg cells. De novo production of miR-181a/b-1^−/−^ tTreg cells was impaired because of altered sensitivity to TCR signals during selection. Generation of Treg cells in the absence of miR-181a/b-1 resulted in elevated expression of CTLA-4, which penetrated into the periphery despite the fact that peripheral WT Treg cells express very low amounts of miR-181a. As a consequence, miR-181a/b-1^−/−^ Treg cells had an increased suppressive capacity.

## Results

### miR-181a/b-1 controls intrathymic Treg-cell development

**Fig 1:**
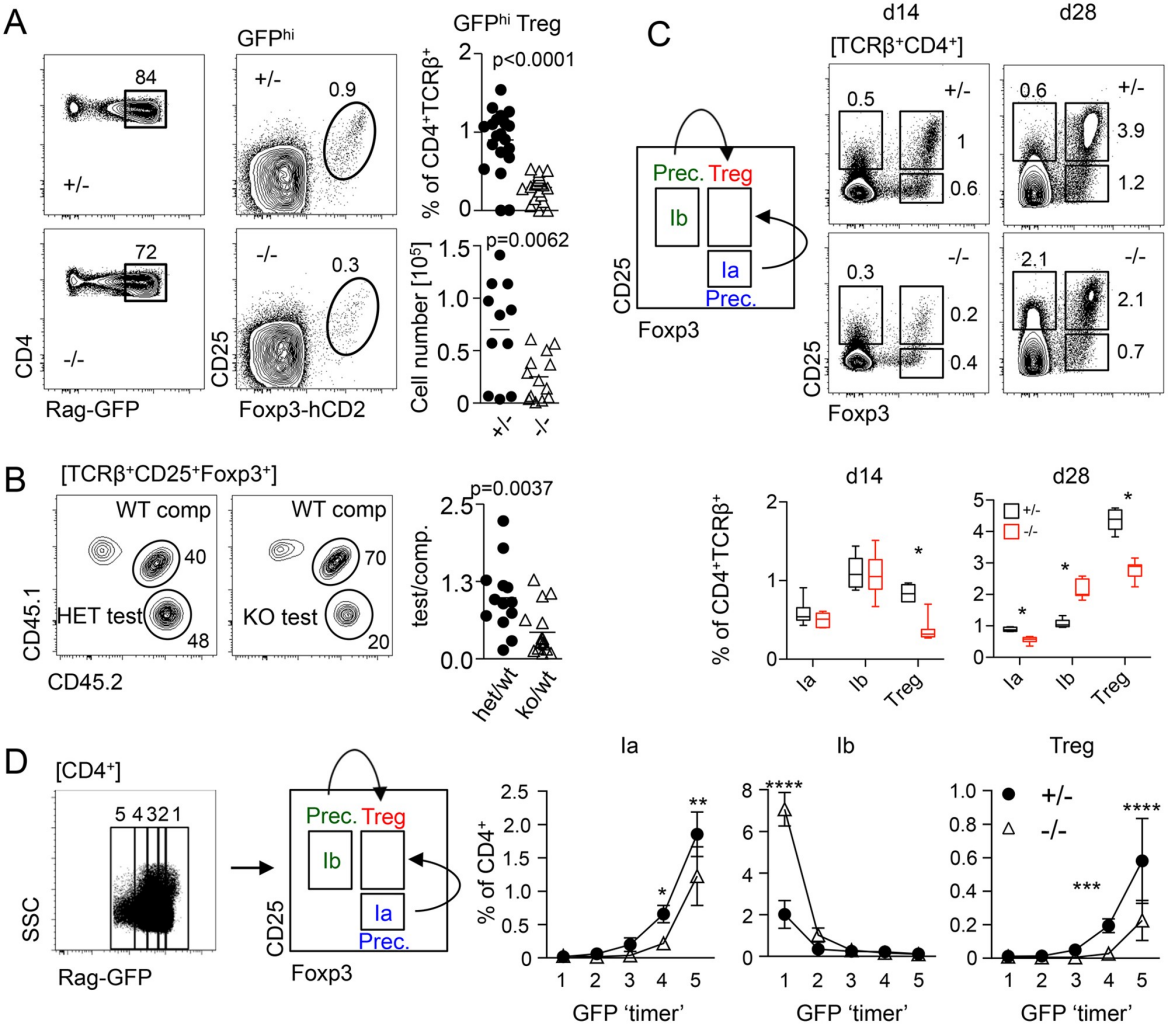
miR-181a/b-1 control intrathymic development of Treg cells. (A) Representative plots, frequencies, and absolute numbers of TCRβ^+^CD4^+^RagGFP^hi^ Treg cells in miR-181–sufficient and deficient mice. Data from 5 independent experiments; each data point represents one mouse. (B) Competitive BM chimeras. BM cells from miR-181a/b-1^+/−^ (het) or miR-181a/b-1^−/−^ (KO) mice (both CD45.2) were mixed in a 1:1 ratio with competitor WT BM cells (CD45.1/2) and injected into lethally irradiated WT recipients (CD45.1). Chimeras were analyzed 12 weeks later for the generation of TCRβ^+^CD4^+^Foxp3^+^ cells in the thymus. Plots are representative of 2 independent experiments with *n* = 8 for each genotype. Graph shows ratio of TCRβ^+^CD4^+^Foxp3^+^ cells within test versus competitor populations. Each data point represents an individual mouse. (C) Frequencies of tTreg cells (TCRβ^+^CD4^+^CD25^+^Foxp3^+^) and their precs TCRβ^+^CD4^+^CD25^−^Foxp3^+^ (Ia) and TCRβ^+^CD4^+^CD25^+^Foxp3^−^ (Ib) on d 14 and 28 after initial induction of Rag1 expression in InduRag1 mice sufficient and deficient for miR-181a/b-1. Depicted data are from 2 independent experiments, with *n* = 1–4 for each genotype and time point analyzed. Below, quantification of the experiment presented in (C). (D) Molecular age of miR-181a/b-1 Treg cells and their precs assessed using *Rag1*^GFP^ reporter mice. Representative plot and gating strategy (left) and quantification (right). Data from 2 independent experiments, *n* = 3–4 for each genotype. Statistical analysis was performed using unpaired Student’s *t* test (A, B), multiple *t* test (C), and two-way ANOVA (D, *p*-values for effect of genotype, **p = 0.0021, ***p = 0.0002 and ****p < 0.001). Numerical values are available in S1 Data. BM, bone marrow; CD, cluster of differentiation; d, day; Foxp3, forkhead box protein P3; GFP, green fluorescent protein; hCD2, human CD 2; InduRag1, inducible recombination-activating gene 1; KO, knockout; miR-181, microRNA-181; prec, precursor; *Rag1*, recombination-activating gene 1; TCR, T-cell receptor; Treg cell, regulatory T cell; tTreg cell, thymic Treg cell; WT, wild type.

First, we tested the hypothesis that miR-181a/b-1 might play a role during Treg-cell development. Using a recombination activating gene 1–green fluorescent protein (*Rag1*^GFP^) knock-in allele to discriminate between nascent and mature thymus-resident or recirculating Treg cells, we found that frequencies and absolute numbers of de novo generated *Rag1*^GFP^-positive Treg cells were reduced by 2- to 3-fold in miR-181a/b-1^−/−^ mice when compared to control (ctrl), indicating that expression of miR-181a/b-1 is required for normal Treg-cell development in the thymus (Fig 1A). Competitive bone marrow (BM) chimeras with 1:1 mixtures of donor cells from wild-type (WT) and miR-181a/b-1^−/−^ mice revealed a disadvantage in Treg-cell generation, but not more immature double-negative and DP as well as CD4SP populations, from thymocytes of miR-181a/b-1^−/−^ origin, indicating that miR-181a/b-1 controls Treg-cell formation in a cell-intrinsic manner (Fig 1B and S1A Fig). In order to test how miR-181a/b-1 influenced developmental progression towards Foxp3^+^CD25^+^ Treg cells, we analyzed inducible Rag1 (InduRag1) miR-181a/b-1^−/−^ mice, in which a wave of T-cell development can be induced by transient initiation of *Rag1* gene expression through a tamoxifen-inducible Cre recombinase (Cre) [30]. At day 7 after Rag1 induction, only a few CD4SP thymocytes were generated, precluding robust analysis of Treg cell development (S1B and S1C Fig). However, we noted reduced frequencies of postselection DP thymocytes as well as postselection CD4SP thymocytes at this time point in miR-181a/b-1^−/−^ mice when compared to ctrls (S1D Fig). These data support the notion that selection processes are altered in the absence of miR-181a/b-1. At day 14 and day 28, frequencies of Treg cells within CD4SP thymocytes were lower in miR-181a/b-1^−/−^ mice when compared to ctrls (Fig 1C). Consistent with findings at steady state, these data indicate that Treg-cell formation is less efficient in the absence of miR-181a/b-1. Next, we analyzed miR-181a/b-1–dependent formation of Foxp3^−^CD25^+^ (prec 1b) or Foxp3^+^CD25^−^ (prec 1a) Treg-cell precursors in the InduRag1 model. At day 14 after induction, both intermediates were present at somewhat similar frequencies in miR-181a/b-1^−/−^ mice when compared to ctrl, whereas at day 28, we observed reduced frequencies of Foxp3^+^CD25^−^ precursors and an accumulation of Foxp3^−^CD25^+^ precursors, suggesting that precursor generation is not restricted by a developmental block (Fig 1C). Taken together, these data indicate that in the absence of miR-181a/b-1, Treg cells are formed with slower kinetics rather than being subject to a defined developmental block. Treg-cell development in the thymus follows a somewhat different course in the absence of a full CD4SP compartment [10]. To account for such differences, we complemented the analysis of InduRag1 mice by taking advantage of the *Rag1*^GFP^ knock-in allele described above to temporally separate Treg-cell development at steady state. Green fluorescent protein (GFP^+^) cells were arbitrarily gated into 5 populations based on different GFP levels, with loss of GFP expression indicating increasing amounts of time since cessation of Rag gene expression, which occurs in DP thymocytes. Frequencies of precursor 1b were elevated in GFP^hi^ cells from miR-181a/b-1^−/−^ mice when compared to ctrls. In contrast, generation of precursor 1a as well as Treg cells was delayed in miR-181a/b-1^−/−^ mice when compared to ctrls (Fig 1D), which is consistent with data obtained in the InduRag1 model. We conclude that loss of miR-181a/b-1 results in an overall delay of Treg-cell formation, which is likely to be initiated prior to the emergence of defined Treg-cell precursors and cannot be compensated for by increased frequencies of Foxp3^–^CD25^+^ precursors.

### TCR signal strength determines miR-181a/b-1–dependent Treg-cell development

**Fig 2:**
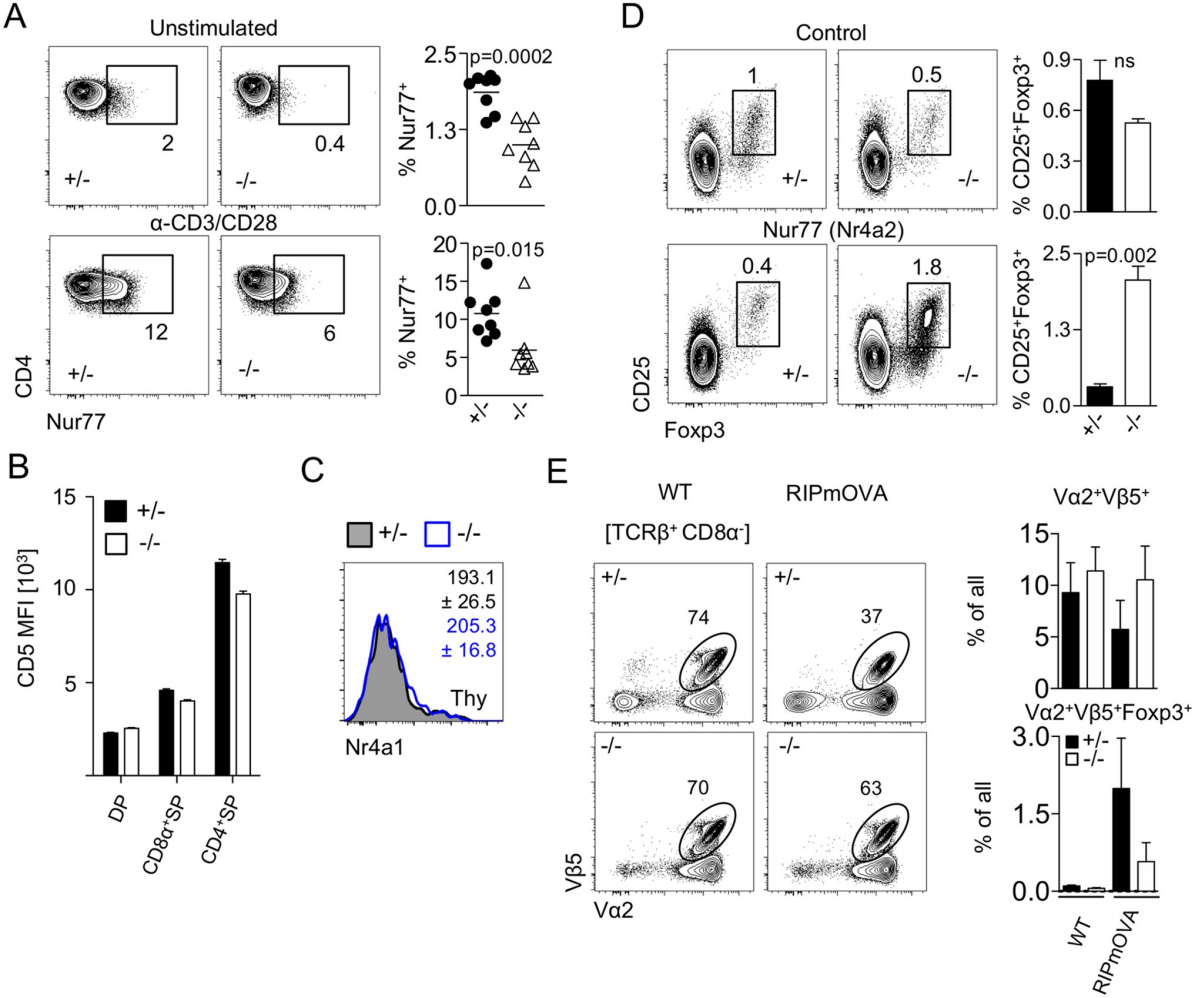
TCR signal strength determines miR-181a/b-1–dependent Treg-cell development. (A) Expression of Nur77 in DP thymocytes in the absence of miR-181a/b-1. Unstimulated or stimulated (3 h, 37 °C, αCD3/CD28 2.5 μg/ml) thymocytes from miR-181a/b-1 het and KO mice were stained for intracellular Nur77. Plots are representative of 2 independent experiments with *n* = 3–4 for each genotype. Graphs show frequencies of Nur77-positive cells for indicated conditions in CD4^+^CD8α^+^ DP thymocytes. (B) Reduced CD5 expression in the absence of miR-181a/b-1 mirrors impaired TCR signaling. Data from 2 independent experiments with *n* = 8–9 per group. (C) RNA flow-cytometry analysis of the expression of *Nr4a1* (encoding Nur77) by TCRβ^+^CD4^+^Foxp3^+^ tTreg cells. Numbers indicate average MFI ± SD. Data from 2 independent experiments with *n* = 2–3 per experiment. (D) Overexpression of Nr4a2 (Nur77 family) rescues development of Treg cells deficient for miR-181a/b-1. LSK cells from OT-II × miR-181a/b-1^+/−^ or OT-II × miR-181a/b-1^–/–^mice were sorted and transduced with a retrovirus expressing a chimeric Nr4a2 molecule in which the Nr4a2 LBD is replaced by that of a mutant human estrogen receptor-α (Nr4a2-ΔLBD-ERT2) or ctrl retrovirus. Cells were injected into lethally irradiated WT recipients, and 7 weeks later, expression of Nr4a2 was induced via tamox administration for 5 consecutive days before analysis. Plots depict frequencies of Treg cells generated from precursor cells transduced with ctrl vector (upper panels) and from precursors transduced with vector carrying inducible Nr4a2 (lower panels). Graphs show summary of the results from 2 independent experiments, with *n* = 3 for each genotype and condition. (E) Less efficient clonal deletion and Treg-cell formation in the absence of miR-181a/b-1. OT-II × RIPmOVA chimeras were generated after lethal irradiation of RIPmOVA recipient mice (ctrl: WT/WT; transgenic: tg/WT) and injection of OT-II × miR-181a/b-1^+/−^ or OT-II × miR-181a/b-1^−/−^ BM cells. Mice were analyzed after 8 weeks. Plots depict frequencies of Vα2^+^Vβ5^+^ OVA-specific donor cells in ctrl and tg recipients, which express OVA during negative selection. Graphs show summary of this analysis (upper panel) and absolute numbers as well as frequencies of Vα2^+^Vβ5^+^Foxp3^+^ Treg cells generated in the presence or absence of miR-181a/b-1 (lower panels). Data are representative of 2 independent experiments with *n* = 6–9 for each recipient/donor combination. Statistical analysis was performed using unpaired Student’s *t* test (A, C, D). Numerical values are available in S1 Data. BM, bone marrow; CD, cluster of differentiation; ctrl, control; DP, double positive; ERT2, tamoxifen-inducible estrogen receptor 2; Foxp3, forkhead box protein P3; LBD, ligand-binding domain; LSK, lineage-negative, stem cell antigen-1-positive, Kit-positive; MFI, mean fluorescence intensity; miR-181, microRNA-181; *Nr4a1*, Nuclear receptor subfamily 4 group A member 1; Nur77, Nuclear hormone receptor NUR/77 encoded by *Nr4a1*; OT-II, ovalbumin-specific MHC class II-restricted alpha beta TCR; OVA, chicken ovalbumin; prec, precursor; RIPmOVA, rat insulin-promoter–driven membrane-bound chicken ovalbumin; SP, single positive; tamox, Tamoxifen; TCR, T-cell receptor; tg, transgenic; Treg cell, regulatory T cell; WT, wild type.

In order to test whether TCR signal strength differed in thymocytes from miR-181a/b-1^−/−^ mice, we assessed expression of Nuclear hormone receptor NUR/77 (Nur77) as a surrogate marker. Of note, miR-181a/b-1^−/−^ DP cells expressed lower levels of Nur77 prior to stimulation when compared to ctrls (Fig 2A). Furthermore, ex vivo stimulation of miR-181a/b-1^−/−^ DP cells failed to induce WT levels of Nur77, together suggesting that miR-181a/b-1^−/−^ DP cells received weaker TCR signals and failed to respond to TCR triggering with the same sensitivity as their WT counterparts (Fig 2A). In order to test whether TCR signaling was impaired prior to Treg-cell generation, we assessed surface expression of CD5, which correlates with TCR signal strength, on thymocyte subsets [31]. At steady state, total DP thymocytes, the majority of which have not undergone selection, displayed similar levels of surface CD5 in the presence and absence of miR-181a/b-1 (Fig 2B). However, CD4SP thymocytes from miR-181a/b-1^−/−^ mice displayed lower surface levels when compared to ctrls. tTreg cells from either genotype expressed similar levels of Nur77 transcripts (Fig 2C), together indicating that miR-181-a/b-1 limits TCR signal strength prior to the emergence of Treg cells.

Next, we took advantage of a system mimicking increased TCR signal strength during Treg-cell development via inducible nuclear translocation of the Nur77 family member Nr4a2 [32]. BM chimeric mice were generated to carry miR-181a/b-1–deficient or miR-181a/b-1–sufficient ovalbumin-specific MHC class II-restricted alpha beta (OT-II) TCR transgenic cells expressing inducible Nr4a2. Transduction with ctrl virus resulted in generation of low frequencies of Treg cells from miR-181a/b-1–sufficient mice, and even fewer Treg cells emerged from miR-181a/b-1^−/−^ donor BM cells (Fig 2D). Upon activation of Nr4a2, frequencies of Treg cells generated from miR-181a^+/−^ donors were slightly, albeit not significantly, reduced, suggesting that activation of Nr4a2 promotes a shift towards clonal deletion. In contrast, in the absence of miR-181a/b-1, Treg-cell development was rescued upon activation of Nr4a2, supporting the hypothesis that limited TCR signal strength accounts for defective Treg-cell differentiation in miR-181a/b-1^−/−^ mice. To corroborate these data, we analyzed chimeric mice generated using OT-II TCR transgenic miR-181a/b-1–sufficient (OT-II-ctrl) or deficient (OT-II-knockout [KO]) donor cells transferred into RIPmOVA recipients. RIPmOVA mice express the cognate antigen for the OT-II TCR in the thymus, resulting in clonal deletion of OT-II TCR transgenic cells as well as generation of low numbers of Treg cells. OT-II-KO>RIPmOVA chimeras showed lower levels of clonal deletion and generated considerably lower numbers of Treg cells when compared to OT-II-ctrl>RIPmOVA chimeras (Fig 2E). OT-II-ctrl>WT as well as OT-II-KO>WT chimeras failed to generate sizeable numbers of OT-II Treg cells and showed no signs of clonal deletion of OT-II thymocytes. We conclude that impaired generation of Treg cells in miR-181a/b-1–deficient mice is due to restricted TCR signal strength during thymic selection.

### Homeostatic expansion generates normal Treg-cell numbers in the periphery of miR-181a/b-1^−/−^ mice

**Fig 3:**
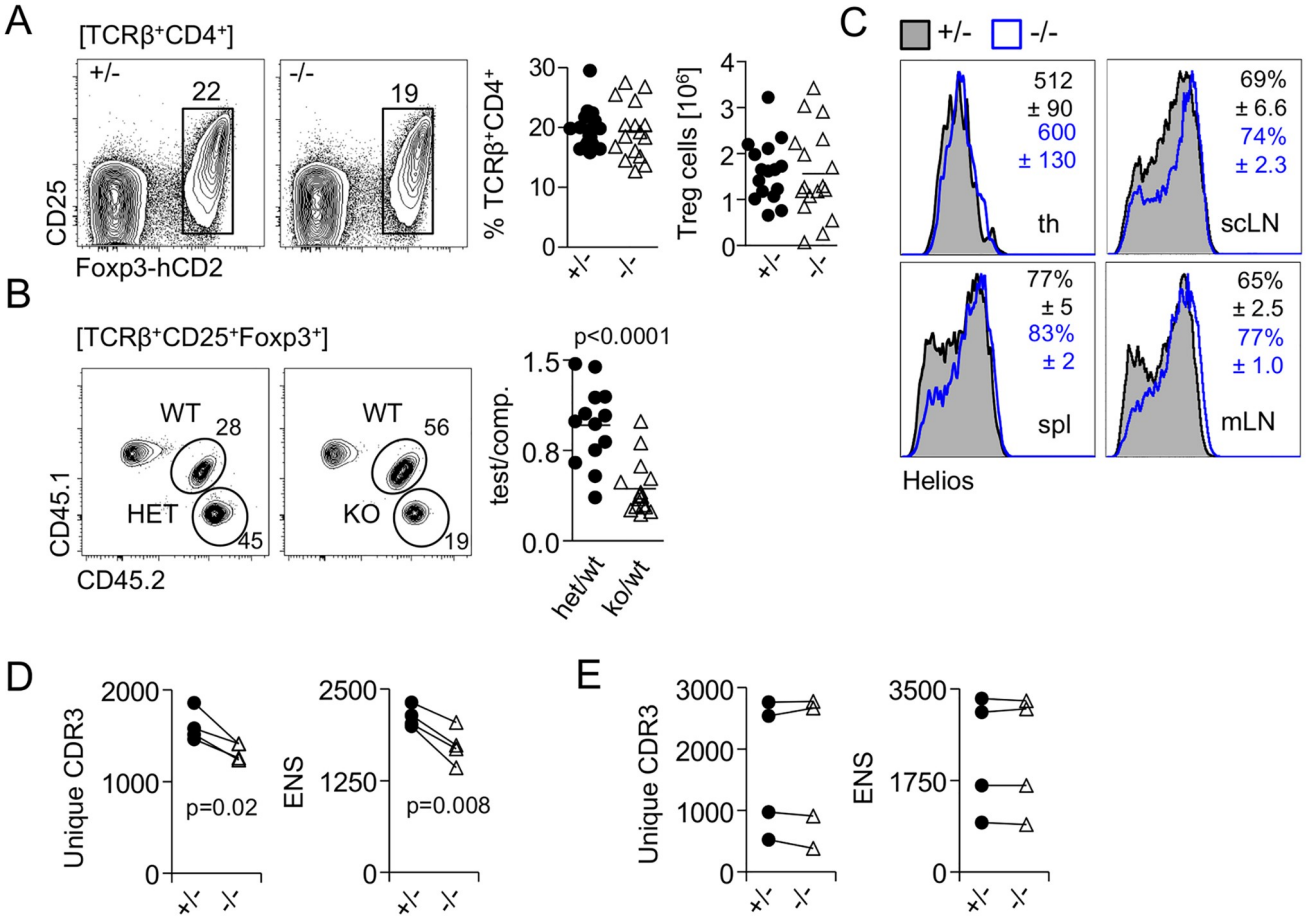
Homeostatic expansion generates normal Treg-cell numbers in the periphery of miR-181a/b-1^−/−^ mice. (A) Frequencies and absolute numbers of splenic Treg cells in Foxp3^hCD2^ reporter mice crossed to miR-181a/b-1 KO mice. Plots are representative of 6 independent experiments. Graph shows summary of all experiments, in which each data point represents one mouse. (B) Competitive BM chimeras. BM cells isolated from miR-181a/b-1^+/−^ (het) or miR-181a/b-1^−/−^ (KO) mice (both CD45.2) were mixed in 1:1 ratio with competitor BM cells, WT (CD45.1/2), and injected into lethally irradiated WT recipients (CD45.1). Chimeras were analyzed 12 weeks after injection for the generation of TCRβ^+^CD4^+^Foxp3^+^ cells in the spl. Plots are representative of 2 independent experiments with *n* = 8 for each genotype. Graph shows ratio of TCRβ^+^CD4^+^Foxp3^+^ cells within tested to competitive populations. Each data point represents an individual mouse. (C) Higher frequencies of Helios-positive cells in peripheral lymphoid organs of miR-181a/b-1^−/−^ mice. Representative histograms and plots from 2 independent experiments (*n* = 6–9 for each genotype) are depicted. Numbers indicated average MFI or frequencies of positive cells, ±SD. Statistical analysis was performed using unpaired Student’s *t* test. (D,E) High-throughput sequencing of TCRα (CDR3 fragment of Vα8–Cα chain) in splenic Treg (D) and tTreg (E) cells sorted from miR-181a/b-1^+/−^ or miR-181a/b-1^−/−^ mice. Left graph shows number of all unique CDR3 sequences (CDR3). Right graphs show ENS calculated for each sample. Depicted are 4 independent sequencing experiments; samples from the same experiment are paired. Statistical analysis was performed using paired Student’s *t* test. Numerical values are available in S1 Data. BM, bone marrow; CD, cluster of differentiation; comp., competitor; ENS, effective number of species; Foxp3, forkhead box protein P3; hCD2, human CD2; KO, knockout; MFI, mean fluorescence intensity; miR-181, microRNA-181; mLN, mesenteric lymph node; scLN, subcutaneous lymph node; spl, spleen; TCR, T-cell receptor; th, thymus; Treg cell, regulatory T cell; tTreg cell, thymic Treg cell; WT, wild type.

To address potential consequences of impaired tTreg-cell development in the absence of miR-181a/b-1, we next determined frequencies of Treg cells in the periphery. We did not observe any differences in frequencies and absolute numbers of peripheral Treg cells in spleens from miR-181a/b-1^−/−^ mice compared to ctrls (Fig 3A). Consistently, frequencies of recirculating or thymus-resident (*Rag1*^GFP^-negative) Treg cells in the thymus were largely unaffected in the absence of miR-181a/b-1, but we observed reduced frequencies of *Rag1*^GFP^-positive recent thymic emigrants (RTEs) among peripheral Treg cells (S2A and S2B Fig). Equilibration of Treg-cell numbers in the periphery can occur through homeostatic expansion of tTreg cells or preferential peripheral induction from naive T cells. Spleens of miR-181a/b-1–sufficient and deficient mice contained comparable frequencies of RTEs (TCRβ^+^Rag1-GFP^+^) cells, which are enriched in peripheral Treg cell precursors (S2B Fig) [33]. Furthermore, CD4^+^CD25^−^ RTEs from miR-181a/b-1^−/−^ mice did not produce more iTreg cells upon transfer into lymphopenic interleukin-7 receptor α gene (*Il7r*)^−/−^ hosts when compared to ctrls, suggesting that Treg-cell induction is not the primary mechanism to equilibrate peripheral Treg-cell numbers in miR-181a/b-1^−/−^ mice (S2C Fig). Chimeric mice generated with 1:1 mixtures of miR-181a/b-1^−/−^ and WT BM showed that miR-181a/b-1^−/−^ Treg cells had a competitive disadvantage in the periphery when WT Treg cells were present, indicating that niche availability permits homeostatic expansion of tTreg cells in miR-181a/b-1^−/−^ mice (Fig 3B). This conclusion was supported by Helios staining and TCR repertoire analysis. Elevated Helios expression has been associated with Treg-cell activation and proliferation [34]. Comparison of tTreg cells from miR-181a/b-1–sufficient and deficient mice showed low and similar expression of Helios between genotypes (Fig 3C). Staining in peripheral lymphoid organs (spleen, subcutaneous lymph node (scLN), and mesenteric lymph node [mLN]) revealed elevated numbers of Helios^+^ Treg cells in miR-181a/b-1^−/−^ mice, indicating that in these mice, more Treg cells are in an activated/proliferative state (Fig 3C).

We predicted that limited de novo generation and increased peripheral expansion resulted in reduced TCR diversity in peripheral Treg cells in the absence of miR-181a/b-1. Comparison of numbers of unique CDR3 sequences as well as calculation of effective number of species as a measure for repertoire diversity showed that TCR diversity in peripheral Treg cells from miR-181a/b-1^−/−^ mice was significantly reduced (Fig 3D and S3A Fig). In contrast, in the thymus, Treg cells from miR-181a/b-1^−/−^ mice displayed comparable TCR diversity as their ctrl counterparts (Fig 3E and S3A Fig). Thus, these data indicate that, as a consequence of less efficient generation, fewer clones egress from the thymus to be available for peripheral expansion.

### Inefficient intrathymic generation of Treg cells results in a post-transcriptional increase in CTLA-4 expression

**Fig 4:**
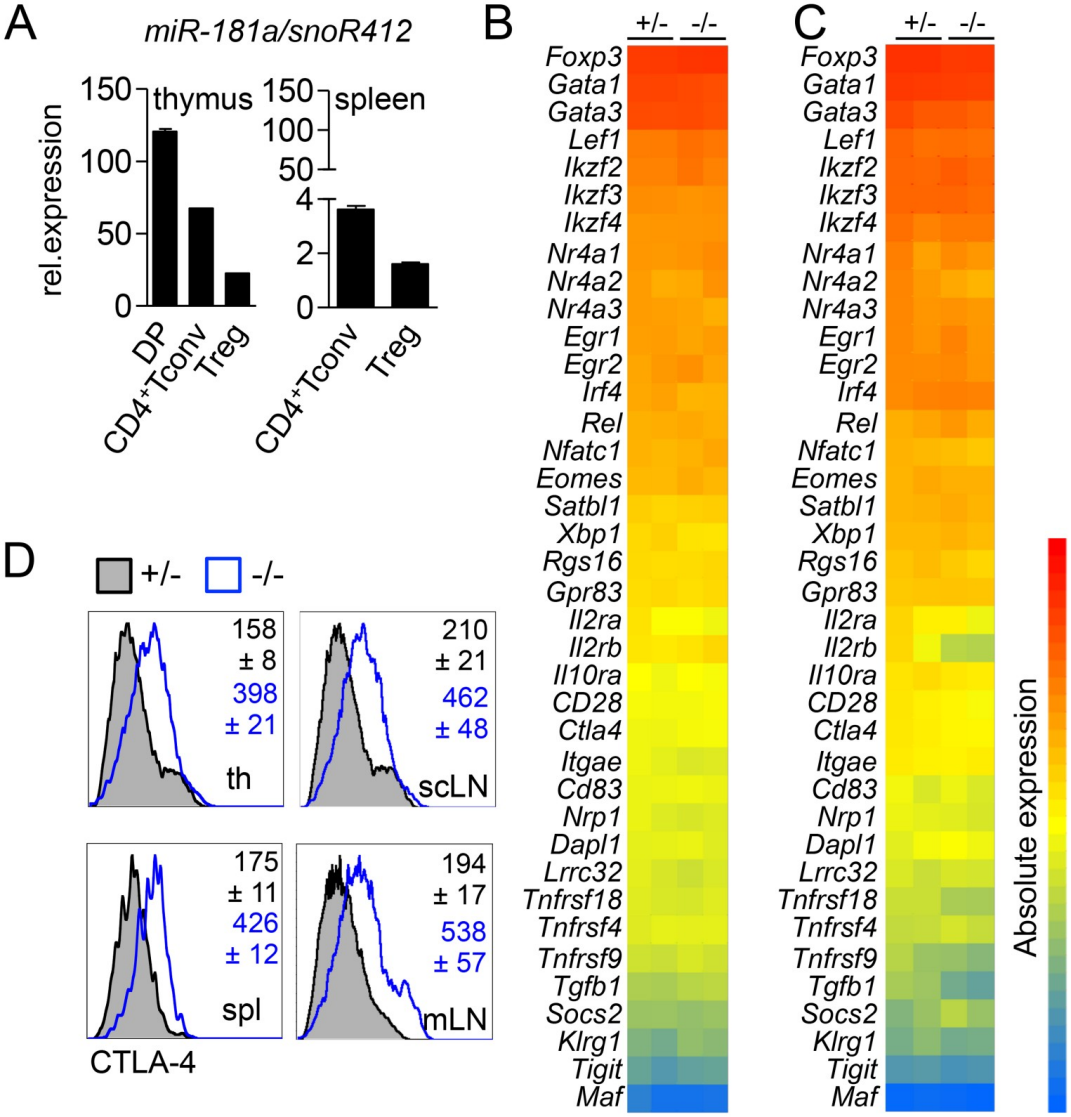
Inefficient intrathymic generation of Treg cells results in post-transcriptional increase in CTLA-4 expression. (A) qRT-PCR analysis of miR-181a expression in sorted thymic populations (left graph) and splenic populations (right graph). Data from 2 independent experiments, with *n* = 6–7 (pool) for each genotype and one experiment for tTreg cells and CD4^+^ Tconv cells, *n* = 7 (pool). Expression of miR-181a was normalized to the expression of housekeeping small RNA, snoR412. Linearized ΔC_T_ values are displayed on the graph. Transcriptional analysis of splenic Treg (B) and tTreg (C) cells. Heat map of signature genes (absolute expression) in miR-181a/b-1^+/−^ or miR-181a/b-1^−/−^ Treg cells. Columns on the heat map represent 2 individual samples from pooled thymi of 5–7 animals for each genotype. (D) Flow-cytometry analysis of CTLA-4 expression in tTreg and peripheral Treg cells. Representative histograms of 2 independent experiments (*n* = 6–9 for each genotype) are depicted. Numbers indicate average MFI ± SD. Numerical values are available in S1 Data. CD, cluster of differentiation; CTLA-4, cytotoxic T-lymphocyte–associated protein 4; *Dapl1*, death-associated protein-like 1; DP, double positive; *Egr*, early growth response gene; *Eomes*, eomesodermin; Foxp3, forkhead box protein P3; *Gata*, GATA-binding factor; *Gpr83*, G-protein-coupled receptor 83; *II2ra*, interleukin-2 receptor alpha; *II2rb*, interleukin-2 receptor beta; *II10ra*, interleukin-10 receptor alpha; *Ikzf*, Ikaros zinc finger; *Irf4*, interferon regulatory factor 4; *Itgae*, integrin alpha E; *Klrg1*, killer cell lectin-like receptor subfamily G member 1; *Lef1*, lymphoid enhancer-binding factor 1; *Lrrc32*, leucine-rich repeat containing 32; *Maf*, musculoaponeurotic fibrosarcoma oncogene homolog; MFI, mean fluorescence intensity; miR-181, microRNA-181; mLN, mesenteric lymph node; *Nfatc1*, nuclear factor of activated T cells c1; *Nrp1*, neuropilin 1; *Nr4a*, Nuclear receptor subfamily 4 group A; qRT-PCR, quantitative reverse-transcription PCR; *Rel*, homolog to the oncogen protein of the reticuloendotheliosis virus strain; *Rgs16*, regulator of G protein signaling 16; *Satbl1*, special AT-rich sequence-binding protein-1; scLN, supraclavicular lymph node; snoR412, small nucleolar RNA 412; *Socs2*, suppressor of cytokine signaling 2; spl, spleen; Tconv, conventional T; *Tgfb1*, transforming growth factor beta 1; th, thymus; *Tigit*, T-cell immunoreceptor with Ig and ITIM domains; *Tnfrsf4*, tumor necrosis factor receptor superfamily member 4; Treg cell, T regulatory cell; tTreg cell, thymic Treg cell; *Xbp1*, X-box binding protein 1.

Next, we assessed whether impaired generation in the thymus altered the phenotype of Treg cells in the absence of miR-181a/b-1. Expression of miR-181a progressively decreases after thymocytes exit the DP stage, with CD4^+^SP thymocytes and thymic Treg cells expressing 2-fold and 15-fold lower levels, respectively (Fig 4A). In the periphery, expression of miR-181a was further reduced to 30-fold and 75-fold lower levels for CD4^+^ conventional T cells (Tconv) and Treg cells, respectively, when compared to DP thymocytes. Peripheral miR-181a/b-1^−/−^ Treg cells showed a virtually identical global gene expression profile to their ctrl counterparts. Notably, this was also the case for key Treg-cell signature genes (Fig 4B). Comparison of transcriptomes of thymic miR-181a/b-1^−/−^ Treg cells with their WT counterparts also revealed no significant differences both globally as well as with regard to Treg-cell signature genes (Fig 4C). Given that miRNAs may also act on a post-transcriptional level, we next assessed expression levels of Treg-cell signature receptors and transcription factors. Peripheral and tTreg cells from miR-181a/b-1^−/−^ mice showed similar expression levels of most surface receptors and transcription factors analyzed as WT ctrls (S4A, S4B and S4C Fig). Notably, we detected strongly increased levels of total CTLA-4 protein in both peripheral and tTreg cells from miR-181a/b-1^−/−^ mice when compared to ctrls (Fig 4D). Although the coding sequence of *Ctla4* mRNA contains putative miR-181 binding sites, direct modulation of CTLA-4 expression by miR-181 could not be observed in luciferase assays (S5A and S5B Fig).

We conclude from these data that thymic generation in the absence of miR-181a/b-1 results in post-transcriptionally controlled up-regulation of CTLA-4 protein in Treg cells while other Treg-cell signature genes remain unaffected. Loss of miR-181a expression in WT peripheral Treg cells suggests that elevated expression of CTLA-4 in these cells is imprinted during development.

### Elevated levels of CTLA-4 in the absence of miR-181a/b-1 are maintained via increased rates of translation

**Fig 5:**
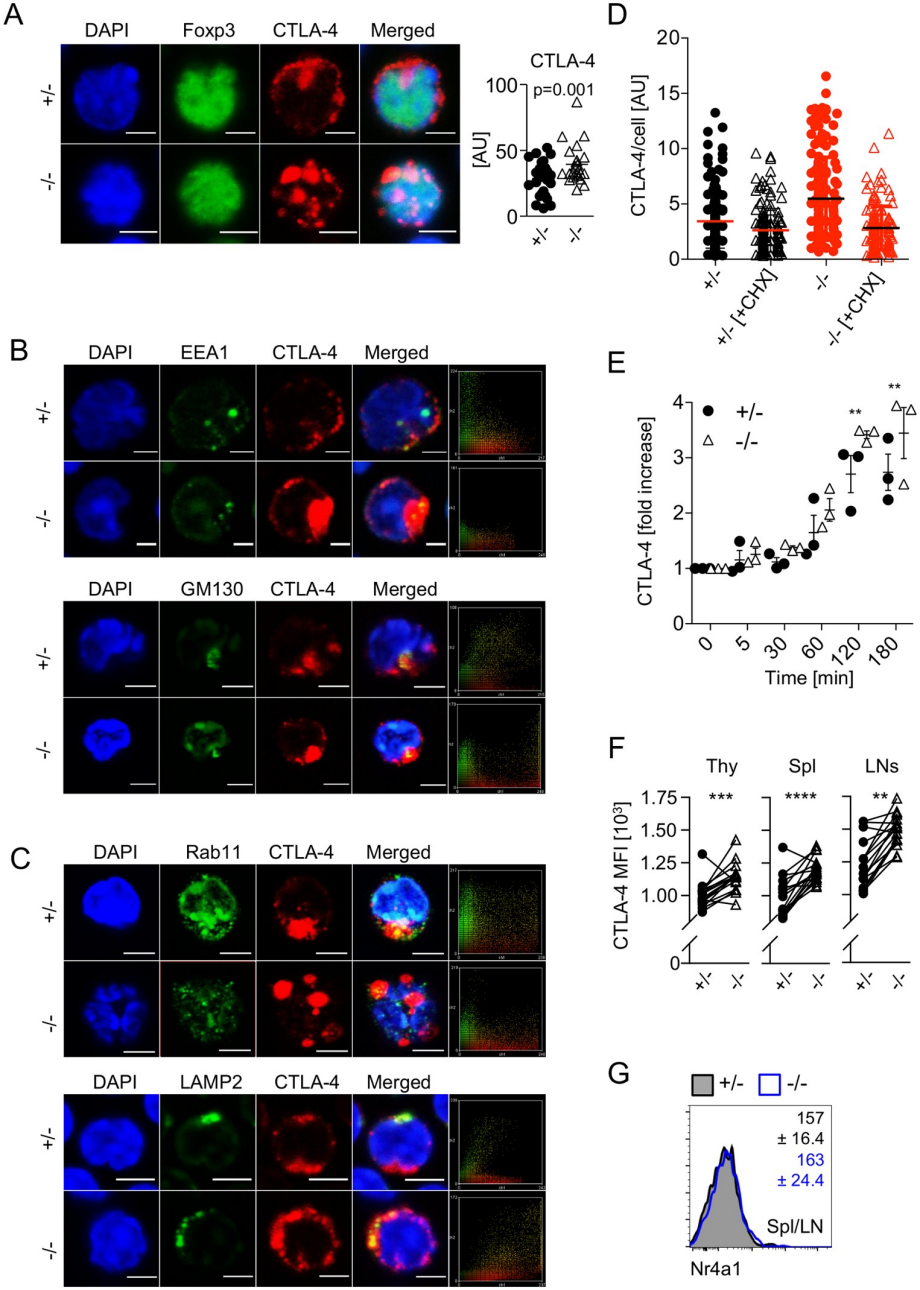
Elevated levels of CTLA-4 in the absence of miR-181a/b-1 are cell-intrinsically maintained via increased rates of translation. (A) Immunofluorescence staining of Treg cells from miR-181a/b-1^+/−^ or miR-181a/b-1^−/−^ mice with antibodies against Foxp3 and CTLA-4. Right panel, quantification of integrated density for CTLA-4 fluorescence normalized to the area of each individual cell. Data are representative of 3 independent experiments with *n* = 3 for each genotype (pooled). Data were analyzed using ImageJ software. DAPI, nuclear staining. Scale bar, 2 μm. (B) Immunofluorescence staining of CD4^+^ T cells from miR-181a/b-1^+/−^ or miR-181a/b-1^−/−^ mice with antibodies against murine CTLA-4, EEA1 (upper panel) and GM130 (lower panel). Data are representative of 3 independent experiments with *n* = 3 (pool). Right panels show colocalization scatter plots. (C) Immunofluorescence staining of CD4^+^T cells from miR-181a/b-1^+/−^ or miR-181a/b-1^−/−^ mice with antibodies against murine CTLA-4, Rab11 (upper panel) and LAMP2 (lower panel). Data are representative of 3 independent experiments with *n* = 3 (pool). Right panels show colocalization scatter plots. Data were analyzed using ImageJ software. DAPI, nuclear staining. Scale bar, 2 μm. (D) Enhanced degradation of CTLA-4 in the absence of miR-181a/b-1. CD4^+^ T cells isolated from spls and subcutaneous LNs of miR-181a/b-1^+/−^ or miR-181a/b-1^−/−^ mice were stimulated for 2 h with αCD3/αCD28 antibodies. In order to monitor protein degradation, CHX was added and incubated with cells for an additional 3 h. Each dot represents an individual cell from 10–12 randomly chosen fields of view. Quantification of integrated density for CTLA-4 fluorescence per cell was quantified using ImageJ software. (E) CD4^+^CD25^+^-enriched T cells were incubated in the presence of an inhibitor of lysosomal degradation, bafilomycin, for indicated time points. Accumulation of CTLA-4 was detected using FACS, and fold increase of MFI was calculated using time point 0 as reference. Data from 3 independent experiments, *n* = 3. (F) Cell-intrinsic up-regulation of CTLA-4 in the absence of miR-181a/b-1. Mixed BM chimeras were generated by mixing BM cells from miR-181a/b-1^+/−^ (CD45.1) and miR-181a/b-1^−/−^ (CD45.2) mice in a 1:1 ratio and injecting them into lethally irradiated WT recipients (CD45.1/2). Chimeras were analyzed 8 weeks after reconstitution for CTLA-4 expression in TCRβ^+^CD4^+^Foxp3^+^ cells from the Thy, spl, and LNs. Plots are representative of 2 independent experiments with *n* = 9 per experiment. Each set of paired data points represents an individual mouse. (G) RNA flow-cytometry analysis of the expression of *Nr4a1* by TCRβ^+^CD4^+^Foxp3^+^ Treg cells from pooled spl and LNs. Numbers indicate average MFI ± SD. Data from 2 independent experiments with *n* = 2–3 per experiment. Statistical analysis was performed using unpaired Student’s *t* test (A), two-way ANOVA (E, *p*-values for effect of genotype, **p = 0.0021) and paired Student’s *t* test (F, *p*-values for effect of each genotype, **p = 0.0021, ***p = 0.0002, and ****p < 0.0001). Numerical values are available in S1 Data. AU, arbitrary unit; BM, bone marrow; CD, cluster of differentiation; CHX, cycloheximide; CTLA-4, cytotoxic T-lymphocyte–associated protein 4; EEA1, early endosome antigen 1; FACS, fluorescence-activated cell scan; Foxp3, forkhead box protein P3; GM130, *cis*-Golgi matrix protein 130; LAMP2, lysosome-associated membrane protein 2; LN, lymph node; MFI, mean fluorescence intensity; miR-181, microRNA-181; *Nr4a*, Nuclear receptor subfamily 4 group A; Rab11, Ras-related in brain protein 11; spl, spleen; TCR, T-cell receptor; Thy, thymus; Treg cell, regulatory T cell; WT, wild type.

In order to understand the underlying mechanisms of how elevated levels of CTLA-4 protein are maintained in peripheral miR-181a/b-1^−/−^ Treg cells, we analyzed its intracellular distribution using confocal microscopy. We confirmed elevated expression of CTLA-4 protein in the absence of miR-181a/b-1 (Fig 5A). Next, we determined intracellular localization of CTLA-4 by costaining for Ras-related in brain protein 11 (Rab11, marking recycling endosomes), lysosome-associated membrane protein 2 (LAMP2, late endosomes), early endosome antigen 1 (EEA1, early endosomes), and *cis*-Golgi matrix protein 130 (GM130, Golgi). We detected no differences in the extent of colocalization of CTLA-4 with early endosomes and the Golgi apparatus in Treg cells from miR-181a/b-1^−/−^ mice compared to ctrls (Fig 5B). However, we noted reduced colocalization of CTLA-4 with recycling endosomes but a marked increase in colocalization with late endosomes in miR-181a/b-1^−/−^ Treg cells when compared to ctrls (Fig 5C).

In order to assess whether aberrant localization of CTLA-4 in miR-181a/b-1^−/−^ Treg cells affected protein degradation, we stimulated Treg cells in the absence or presence of the translation inhibitor cycloheximide. Inhibition of translation for 2 h reduced CTLA-4 protein to similar levels in miR-181a/b-1^−/−^ Treg cells and ctrls (Fig 5D). Given the higher protein levels when translation is active, these data imply that degradation rates of CTLA-4 are higher in the absence of miR-181a/b-1, which is consistent with its preferential localization in late endosomes rather than recycling endosomes. Furthermore, these data predict that if protein degradation is intact, elevated levels of CTLA-4 protein arise as a result of increased rates of protein translation. To test this prediction, we assessed accumulation of CTLA-4 protein in Treg cells in the presence of bafilomycin, an inhibitor of lysosomal protein degradation, in vitro. Over the course of 3 hours, miR-181a/b-1^−/−^ Treg cells accumulated significantly more CTLA-4 protein when compared to their WT counterparts (Fig 5E). Together, these data indicate that elevated levels of CTLA-4 in peripheral Treg cells in the absence of miR-181a/b-1 are due to increased rates of translation. Increased protein translation in the absence of altered mRNA levels may be induced by loss of miRNAs other than miR-181a/b-1. To test this possibility, we performed small RNA sequencing (small RNAseq) of miR-181a/b-1–sufficient and deficient peripheral Treg cells. Consistent with the overall small changes in the transcriptome, we identified 4 miRNAs (miR-15b, miR-150, miR-342, and lethal (let)-7g) that were moderately down-regulated in Treg cells from miR-181a/b-1^−/−^ mice (S5C and S5D Fig). However, in silico analysis of *Ctla4* mRNA using Targetscan7 and RNA22 provided no evidence for the existence of either canonical or noncanonical binding sites for any of these miRNAs, suggesting that elevated protein levels of CTLA-4 are not caused by reduced miRNA expression. Next, we assessed whether peripheral expansion contributed to sustained expression of CTLA-4 in peripheral Treg cells. To this end, we generated mixed BM chimeras and analyzed CTLA-4 levels on miR-181a/b-1^−/−^ and WT Treg cells isolated from the same mice. CTLA-4 levels were consistently higher in miR-181a/b-1–deficient Treg cells despite competition by their WT counterparts, indicating that CTLA-4 levels are regulated cell-intrinsically and do not depend on peripheral expansion (Fig 5F). Finally, we tested whether alterations in tonic TCR signaling might result in elevated expression of CTLA-4. Freshly isolated peripheral Treg cells from miR-181a/b-1^−/−^ and WT mice expressed comparable levels of Nr4a1 mRNA, suggesting that tonic signaling through the TCR is similar (Fig 5G). Taken together, these findings further support the hypothesis that miR-181a/b-1–dependent ctrl of CTLA-4 expression is elicited in the thymus and subsequently sustained in the periphery.

### Treg cells from miR-181a/b-1^−/−^ mice display increased suppressive capacity

**Fig 6:**
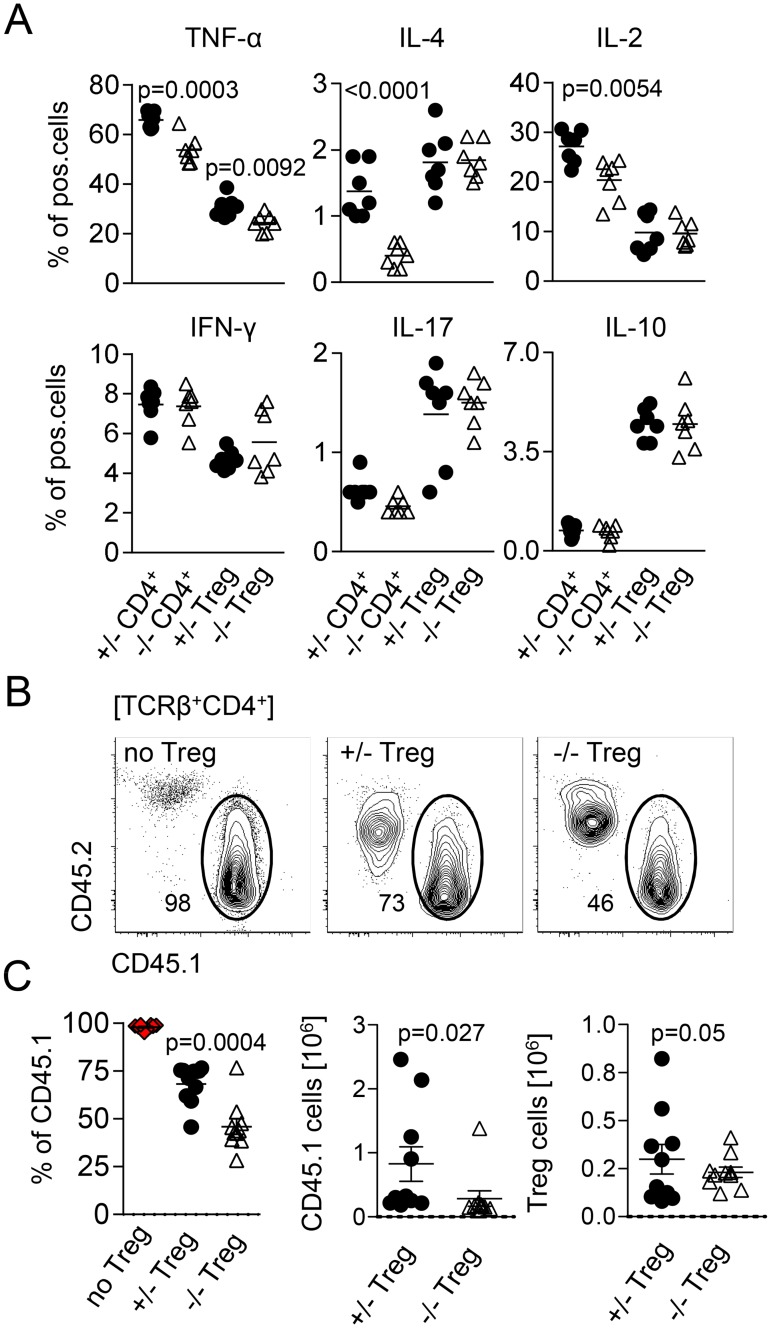
Treg cells from miR-181a/b-1^−/−^ mice display increased suppressive capacity. (A) Production of cytokines by splenic CD4^+^ T cells and Treg cells after stimulation with PMA/ionomycin. Graphs represent quantification of the data from 3 independent experiments, *n* = 4–5 for each genotype. (B) In vivo suppression of homeostatic proliferation in lymphopenic Rag^−/−^ mice by miR-181a/b^+/−^ and miR-181a/b^−/−^ Treg cells. Depicted is expansion of Tconv in the absence (no Treg cells) and presence of miR-181a/b-1^+/−^ or miR-181a/b-1^−/−^ Treg cells 14 days after coinjection of T cells. (C) Quantification and absolute numbers of recovered Tconv cells and Treg cells. Data are representative of 2 independent experiments, with *n* = 7–8 (recipient mice). Statistical analysis was performed using unpaired Student’s *t* test. Numerical values are available in S1 Data. CD, cluster of differentiation; IFN-γ, interferon gamma; IL, interleukin; miR-181, microRNA-181; PMA, phorbol 12-myristate 13-acetate; pos., positive; Tconv, conventional T; TCR, T-cell receptor; TNF, tumor necrosis factor; Treg cell, regulatory T cell.

In order to test the functional consequences of elevated levels of CTLA-4 in miR-181a/b-1^−/−^ Treg cells, we assessed their suppressive capacity. First, loss of miR-181a/b-1 resulted in reduced levels of tumor necrosis factor (TNF)α, IL-4, and IL-2 by conventional CD4^+^ T cells (Fig 6A). In contrast, expression of IL-17 and IL-10 remained unaffected. Whereas levels of TNFα were also reduced in miR-181a/b-1^−/−^ Treg cells, we did not observe additional significant miR-181a/b-1–dependent alterations in cytokine production by Treg cells or CD8^+^ T cells (Fig 6A and S6A Fig). Such alterations in cytokine profiles might be due to cell-intrinsic effects or due to modulation of Treg-cell suppressive capacity. Therefore, we next assessed in vivo suppressive capacity of Treg cells by transfer of congenically marked 1:1 mixtures of miR-181a/b-1–sufficient or miR-181a/b-1–deficient Treg cells (CD45.2) and conventional T cells (CD45.1) into *Rag1*^−/−^ recipients. Suppression of lymphopenia-driven expansion of Tconv cells is dependent on CTLA-4 [35]. At 14 days after transfer, homeostatic expansion of Tconv cells was assessed. We observed lower frequencies of Tconv cells (CD45.1) in spleens of mice co-transferred with miR-181a/b-1^−/−^ Treg cells when compared to those co-transferred with miR-181-a/b-1–sufficient Treg cells (Fig 6B). This reduction in frequency was reflected by lower absolute numbers of Tconv cells recovered in the presence of miR-181a/b-1^−/−^ Treg cells, whereas absolute numbers of Treg cells recovered were similar in both conditions (Fig 6C). Together, these data indicate that in the absence of miR-181a/b-1, Treg cells have a stronger capacity to suppress lymphopenia-driven expansion of Tconv cells in vivo. Of note, we did not observe significant alterations in suppressive capacity of miR-181a/b-1^−/−^ Treg cells in vitro (S6B Fig). Taken together, our data indicate that miR-181a/b-1 controls intrathymic Treg cell development in a TCR-dependent manner. Impaired Treg-cell development in the absence of miR-181a/b-1 is associated with post-transcriptional up-regulation of CTLA-4, which penetrates into the periphery and results in increased suppressive capacity. Low levels of miR-181a in peripheral WT Treg cells suggest that the effects of loss of miR-181a/b-1 are imprinted during intrathymic development.

## Discussion

Here, we demonstrated that intrathymic generation of Treg cells depends on miR-181a/b-1 via establishing signaling thresholds to adequately respond to strong TCR signals. In the absence of miR-181a/b-1, de novo generation of Treg cells was attenuated and resulted in Treg cells expressing elevated levels of CTLA-4. Homeostatic expansion resulted in a completely filled peripheral Treg-cell compartment while CTLA-4 levels remained elevated via a post-transcriptional mechanism, resulting in Treg cells with increased suppressive capacity.

Treg cells develop from CD4SP thymocytes through two possible intermediates, Foxp3^−^CD25^+^ and Foxp3^+^CD25^−^ [8,11]. It has been suggested that generation of these precursors occurs through a TCR-dependent step, whereas further maturation into mature Foxp3^+^CD25^+^ Treg cells is dependent on the cytokines IL-2 and IL-15 [8,10,11]. Analysis of InduRag1 mice as well as a *Rag1*^GFP^ virtual timer indicated that miR-181a/b-1 predominantly affects formation of Foxp3^+^CD25^−^ precursors, whereas Foxp3^−^CD25^+^ are more frequent in miR-181a/b-1–deficient mice. Nevertheless, these precursors cannot compensate for the partial loss of Foxp3^+^CD25^−^ precursors, suggesting that the major route of Treg-cell development is through the latter. Indeed, it has been shown that in WT mice, only approximately 20% of CD4^+^Foxp3^−^CD25^+^ cells ultimately give rise to Treg cells [8,10]. Treg-cell development via Foxp3^−^CD25^+^ intermediates predominantly occurs in double transgenic mouse lines expressing a transgenic TCR plus its cognate antigen [9]. Furthermore, these cells express higher levels of a Nur77^GFP^ reporter than either Foxp3^+^CD25^−^ precursors or mature Treg cells [10]. Thus, it has been suggested that Foxp3^−^CD25^+^ intermediates arise at the extreme end of the TCR affinity spectrum and might increase in frequency by an influx of cells otherwise targeted for clonal deletion. Accordingly, reduction in MHCII levels on thymic epithelial cells in a monoclonal system diverted thymocytes from clonal deletion into the Treg cell lineage [16]. TCR repertoire analyses and autoreactivity suggest that TCR signal strength required for tTreg-cell generation overlaps both with that of positively selected thymocytes as well as that of cells normally undergoing clonal deletion [3,13,14]. Rescue experiments performed in this study agree with both non-mutually exclusive models. Fewer donor-derived miR-181a/b-1^−/−^ OT-II Treg cells developed in RIPmOVA antigen transgenic mice compared to WT OT-II Treg cells. Concomitantly, clonal deletion of miR-181a/b-1^−/−^ OT-II cells was also impaired in RIPmOVA mice, suggesting that in this particular paired TCR–antigen model, TCR signal strength is reduced through loss of miR-181a/b-1 to limit both Treg-cell formation and clonal deletion. Conversely, induced expression of Nr4a2 promoted Treg-cell production in the absence of miR-181a/b-1 but resulted in somewhat limited production of Treg cells in the presence of miR-181a/b-1, suggesting that in the latter case, clonal deletion might be favored over Treg-cell development.

Intrathymic development of Treg cells depends on CD28-mediated costimulatory signals [36]. Thus, it might be possible that elevated expression of CTLA-4 by Treg cells in the thymus contributes to impaired development. Costimulation via CD28 is required for efficient generation of Foxp3^−^CD25^+^ Treg-cell precursors but less so during later Treg-cell development, suggesting that CD28 signaling protects thymocytes from clonal deletion [36–39]. Loss of CD28 signaling does not result in export of autoreactive cells into the periphery, indicating that it does not simply act as an amplifier of TCR signal strength [39]. Consistently, loss of CD28-mediated costimulation and loss of miR-181a/b-1 generate phenotypically distinct developmental defects, also supporting the notion that elevated levels of CTLA-4 in miR-181a/b-1^−/−^ Treg cells are a consequence rather than cause of inefficient generation of Treg cells in these mice.

Consequences of altered TCR signal strength in the thymus have been previously analyzed in mice carrying hypomorphic mutations of key signal mediators, such as zeta-chain–associated kinase of 70 kD (Zap-70), or reduced numbers of immunoreceptor tyrosine-based activation motifs (ITAMs) within CD3ζ molecules [40–42]. Collectively, these studies showed that alterations in TCR signal affected positive and negative selection as well as Treg-cell formation, albeit in a manner that is not easily predictable. Thus, these data indicate that the quantitative relationship between proximal TCR signaling and effcient thymic selection needs to be tightly balanced. Furthermore, mutations characterized in these studies equally affect T-cell activation and tonic signaling in the periphery, precluding analysis of developmental consequences of altered TCR signaling exclusively occurring in the thymus.

In contrast, expression levels of miR-181a/b-1 in peripheral Treg cells are very low and should therefore allow WT-like levels of tonic TCR signaling. Notably, peripheral TCR signaling controls Treg-cell homeostasis and helps to maintain functional Treg cells [43,44]. For instance, in the absence of peripheral TCR expression, levels of CTLA-4 protein are reduced, and suppressive capacity is compromised [44].

Our data suggest that alterations in thymic selection caused by the absence of miR-181a/b-1 have long-term impact and are translated to increased suppressive activity of peripheral Treg cells. We therefore propose that the developmental legacy of TCR signal strength during agonist selection determines Treg-cell function in the periphery. Thus, altered TCR thresholds during selection might affect a Treg cell’s responsiveness to tonic signaling. Similar observations have previously been reported for both CD4 and CD8 Tconv cells [45,46]. The avidity of positively selecting self-peptides and thus strength of the TCR signal during selection determines the outcome of a T-cell immune response even of T cells recognizing the same foreign antigen with an identical avidity [45]. In contrast to Treg cells, differential reactivity to self-peptide by CD8 Tconv cells was accompanied by clear differences in gene expression profiles [46]. Although in Tconv cells, the capacity for tonic signaling in the periphery contributes to distinct responsiveness to pathogens, thymically predetermined levels of the feedback regulator of TCR signaling CD5 are likely to help control tonic signals [45]. Thus, the quality of protective T-cell responses as well as Treg-cell mediated suppression appear to be preset during thymic selection.

How TCR signal strength during thymic agonist selection confers long-term changes in CTLA-4 protein expression remains unclear. Our study supports a model in which expression of CTLA-4 is cell-intrinsically sustained in the periphery through a post-transcriptional mechanism controlling its translation rate. Translational control can be exerted at multiple different levels, including changes in mRNA composition through alternative splicing and miRNAs as well as RNA binding proteins. Given the lack of umambiguous evidence for one of these mechanisms being predominant, our data suggest that multiple factors may act in concert to control CTLA-4 protein. Tight control of CTLA-4 expression is likely to be paramount for tuning suppressive capacity of Treg cells.

Our study establishes miR-181a/b-1 as a central regulator of agonist-selected αβT cells. Earlier studies showed that miR-181a/b-1 is critical for the development of innate-like T cells expressing semi-invariant TCRs, such as iNKT cells and MAIT cells (but not their γδTCR-expressing counterparts) [25,26,47]. Here, we demonstrated that the role of miR-181a/b-1 can be extended to highly diverse polyclonal T-cell populations. This finding was not anticipated because it might be expected that a shift in integrated signal strength might be compensated for by a shift in the repertoire. Such compensation might partly explain why the effect of miR-181a/b-1 deletion on Treg cells is somewhat milder when compared to other agonist selected T cells. Finally, based on the dramatic down-regulation of miR-181a after the DP stage, our study implies that the lineage fate decision to become a Treg cell manifests itself early during selection.

## Materials and methods

### Ethics statement

All experiments were performed in accordance with German law on care and use of laboratory animals or with the institutional and ethical guidelines of the University of Edinburgh and have been approved by the Niedersächsisches Landesamt für Verbraucherschutz und Lebensmittelsicherheit (LAVES) (33.12-42502-04-08-1480; 11/0533; 12/0869; 13/1224; 15/1846), the Regierungspräsidium Darmstadt (V54–19c20/15–FU/1119; FU/1155; FU/1159; FU/1178) or under a project license granted by the UK Home Office (PPL60_4510 procedure 19b 5), respectively. Animals were killed by CO_2_ inhalation, in some cases followed by cervical dislocation.

### Mice

MiR-181a/b-1^−/−^ and miR-181a/b-1^+/−^ mice (B6.Mirc14^tm1.1Ankr^) were generated as described in [26] and bred at the Hannover Medical School and Goethe University, Frankfurt, Germany. OT-II mice (B6.Cg-Tg(TcraTcrb)^425Cbn^/J) were purchased from The Jackson Laboratory, crossed with miR-181a/b-1^−/−^ and miR-181a/b-1^+/−^ mice, and bred at the Hannover Medical School. RIPmOVA mice (C57BL/6-Tg(Ins2-TFRC/OVA)^296Wehi/Wehi^J) were purchased from The Jackson Laboratory and crossed with B6 CD45.1. F1 mice (RIPmOVA CD45.1/2) were bred at the Hannover Medical School. C57BL/6J mice (CD45.2), B6.SJL-*Ptprc*^*a*^*Pepc*^*b*^/BoyJ mice (termed “B6 CD45.1” throughout this manuscript), and IL-7Rα–deficient mice (B6.129S7-Il7r^tm1Imx^/J) were purchased from Charles River or The Jackson Laboratory or bred at the animal facility of Hannover Medical School. (C57BL/6J × B6 CD45.1) F1 mice (CD45.1/CD45.2 heterozygous) and IL-7Rα^−/−^ CD45.1 mice were bred at the animal facility of Hannover Medical School. InduRag1^fl/fl^ × Rosa26-CreERT2 mice (termed InduRag1 here) were obtained by crossing InduRag1^fl/fl^ mice with Rosa26-CreERT2 mice (kindly provided by Prof. Anton Berns, The Netherlands Cancer Institute, Amsterdam, The Netherlands) and bred at the Helmholtz Centre for Infection Research [30]. These mice were crossed to miR-181a/b-1^−/−^ (InduRag1 × miR-181a/b-1^−/−^) and miR-181a/b-1^+/−^ (InduRag1 × miR-181a/b-1^+/−^) mice bred and maintained at the Helmholtz Centre for Infection Research in Braunschweig. Foxp3^hCD2^ × Rag1^GFP^ mice were obtained by crossing Foxp3^hCD2^ mice [48] with Rag1^GFP^ mice [49] (kindly provided by Nobuo Sakaguchi, Kumamoto, Japan). These mice were crossed to miR-181a/b-1^−/−^ and miR-181a/b-1^+/−^ mice and bred at the Helmholtz Centre for Infection Research in Braunschweig. *Rag1*^−/−^ mice and B6 CD45.1 mice used in the in vivo suppression assay were bred and maintained at the University of Edinburgh. All mice were used between 7–12 weeks of age and were maintained under specific-pathogen–free conditions.

### In vivo induction of Treg-cell development

Treg-cell development was induced in 6- to 8-week–old InduRag1 × miR-181a/b-1^−/−^ and InduRag1 × miR-181a/b-1^+/−^ mice by oral administration of tamoxifen (Ratiopharm, Ulm, Germany) in corn oil (Sigma Aldrich, St. Louis, MO, USA), 0.6 mg/400 μl/mouse every 4 days. To ensure proper tamoxifen solubility, in the first step, it was dissolved in 100% ethanol prewarmed to 55 °C and later in prewarmed corn oil. Before each oral administration, the mixture was always prewarmed to 37 °C.

### Competitive BM chimeras

B6 CD45.1^+^ recipient mice were lethally irradiated (9 Gy). Donor BM from both WT (CD45.1/2^+^) and miR-181a/b-1^+/−^ or miR-181a/b-1^−/−^ (CD45.2^+^) mice mixed at a 1:1 ratio was injected into the lateral tail vein within 24 h postirradiation (a total of 4 × 10^6^ cells/mouse). Mice were provided with antibiotic-containing water and were housed in sterile microisolator cages. Analysis of BM chimeras was performed 8–12 weeks after transplantation.

### Overexpression of Nr4a2 in BM chimeras

BM cells were isolated from the tibia and femur of age-matched (7–10 weeks) OT-II × miR-181a^−/−^ and OT-II × miR-181a^+/−^ mice. First, lineage-negative (lin^−^) cells were enriched by incubation with the cocktail of purified rat-anti-mouse antibodies (CD19 [1D3], CD11b [M1/70], Gr-1 [RB6-8C5], NK1.1 [PK136], Ter-119, CD4 [RM4.4], and CD8α [53–6.7]; all from eBioscience, Thermo Fisher Scientific, Waltham, MA, USA) followed by incubation with sheep-anti-rat dynabeads (Dynal; Invitrogen, Carlsbad, CA, USA) and magnetic separation. Cells were stained with a phycoerythrin (PE)-Cy7–conjugated cocktail of lin antibodies (CD19 [6D5], CD11b [M1/70], Gr-1 [RB6-8C5], NK1.1 [PK136], Ter-119, CD4 [GK1.5], and CD8α [53–6.7]; all from eBioscience) and Sca-1 (E13–161.7) PacificBlue, c-kit (CD117) allophycocyanin (APC), and sorted as lin^−^Sca-1^+^c-kit^+^ to 98% purity. Cells were cultured overnight at a density of 5 × 10^4^ cells/100 μl (on 96-well plate) with mouse IL-6 (20 ng/ml), mouse IL-7 (25 ng/ml), mouse Flt-3L (25 ng/ml), and mouse SCF (50 ng/ml; all obtained from PeproTech, Rocky Hill, NJ, USA), in DMEM containing 10% FBS. Next day, cells were infected by centrifugation at 700 g for 45 min at 32 °C on retronectin-coated plates (50 μg/ml, 4 °C overnight) loaded (3× 100 μl/well, 2,000 g, 20 min at 32 °C) with Nr4a2-ΔLBD-ERT2–expressing retrovirus or ctrl retrovirus (both carrying Thy1.1 tag detectable by flow ctometry) in the presence of polybrene (8 μg/ml) and the abovementioned cytokines. Fresh medium was supplemented after 24 h. After 48 h, cells were collected, and transduction efficacy (frequency of Thy1.1^+^ cells) was determined. Routinely, it was approximately 60%, and thus cells were later sorted to 100% of Thy1.1^+^. B6 CD45.2^+^Thy1.2^+^ recipient mice were lethally irradiated (9 Gy). Sorted cells were intravenously injected (1 × 10^5^/recipient). Mice were provided with antibiotic-containing water and were housed in sterile microisolator cages.

Six weeks after injection, mice were orally administered 0.6 mg/400 μl of tamoxifen (Ratiopharm) dissolved in corn oil (Sigma Aldrich) per recipient mouse for 5 consecutive days. Tamoxifen preparation was performed as for induction of Treg-cell development in InduRag1 mice. Twelve hours after the final administration, mice were analyzed.

### Intracellular staining of Nur77 in thymocytes

Thymocytes isolated from miR-181a^+/−^ and miR-181a^−/−^ mice were left untreated or were stimulated for 3 h at 37 °C in the presence of plate-bound αCD3 (17A2, 2.5 μg/ml) and soluble αCD28 (37.51, 2.5 μg/ml). Next, cells were stained for surface CD4 and CD8α and intracellular Nur77 with intracellular staining buffer set (eBioscience).

### RIPmOVA × OT-II BM chimeras

BM cells were isolated from the tibia and femur of age-matched (7–10 weeks) OT-II × miR-181a/b-1^−/−^ and OT-II × miR-181a/b-1^+/−^ mice. Red blood cells were lysed, and cells were counted and injected (5 × 10^6^/recipient) into the lateral tail vein of lethally (2 × 4.5 Gy) irradiated RIPmOVA (CD45.1/2^+^) recipient mice. Mice were provided with antibiotic-containing water and were housed in sterile microisolator cages. Analysis of BM chimeras was performed 8–12 weeks after transplantation.

### Flow cytometry and cell sorting

Monoclonal antibodies specific for CD4 (RM4.4, GK1.5), CD8α (53–6.7), CD25 (PC61.5, eBio7D4 for sort), CD24 (M1/69), CD27 (LG.3A10), CD28 (37.51), CD103 (M290), CD122 (TM-β1), CD127 (A7R34), Gr-1 (RB6-8C5), erythroid cell marker (Ter-119), CD19 (1D3, 6D5), CD11b (M1/70), CD45.1 (A20), CD45.2 (104), CD117 (c-kit) (2B8, ACK2), Sca-1 (E13–161.7), NK1.1 (PK136), CD11c (N418), TCRβ (H57-597), Foxp3 (MF23, FJK-16s), human CD2 (RPA-2.10), Qa2 (1-1-2), Nur77 (12.14), Vβ5.1/5.2 TCR (MR9-4), Vα2 TCR (B20.1), CTLA-4 (UC10-4B9, UC10-4F10-11), Helios (22F6), TGF-βR (RI/ALK-5), GITR (DTA-1), PLZF (Mags.21F7), Gata3 (TWAJ), Egr2 (erongr2), Irf4 (3E4), c-Rel (1RELAH5), and KLRG1 (2F1) were used as biotin, PacificBlue, fluorescein isothiocyanate (FITC), Alexa Fluor 488, PE, peridinin chlorophyll protein-Cy5.5 (PerCP-Cy5.5), PE-Cy7, APC, APC-Cy7, APC-eFluor780, Brilliant Violet 421, eFluor450, and Alexa Fluor 647 conjugates. Antibodies were purified from hybridoma supernatants using standard procedures or were purchased from eBioscience, BD Biosciences (San Jose, CA, USA), BioLegend (San Diego, CA, USA), or R&D Systems (Minneapolis, MN, USA). For intracellular stainings, an intracellular staining buffer set and a Foxp3/transcription factor staining buffer set (both eBioscience) were used according to the manufacturer’s protocol. RNA flow cytometry was performed using the PrimeFlow system (Thermo Fisher Scientific). Cells were stained with Nr4a1-AF647. Samples were acquired on LSRII (BD Biosciences) cytometers and sorted on a FACS Aria II (BD Biosciences). Data were analyzed with FlowJo software, v.9.4.9 (Tree Star, Ashland, OR, USA). For analysis, dead cells and debris were excluded by gating of forward and side scatter. Sorted cells were of 98% or higher purity, as determined by reanalysis.

### In vivo Treg-cell induction assay

RTEs depleted of Treg cells were sorted as *Rag1*^GFP+^CD4^+^CD8α^−^CD25^−^ from the spleens of Foxp3^hCD2^ × Rag1^GFP^ × miR-181a/b-1^−/−^ and Foxp3^hCD2^ × Rag1^GFP^ × miR-181a/b-1^+/−^ mice. 5 × 10^5^ cells were injected into the lateral tail vein of lymphopenic IL-7Rα^−/−^ (CD45.1) recipients. Spleens, peripheral lymph nodes (pLNs: inguinal, brachial, axiliary, and cervical), and mLN were analyzed for the induction of Foxp3 within donor population (CD45.2) 28 days later.

### In vitro suppression assay

Splenic antigen-presenting cells (CD45.1^+^) were purified using sheep-anti-rat magnetic beads and depletion of CD19, CD3, NK1.1, and Gr-1–positive cells as described above. Cells were plated on a U-bottom–shaped 96-well plate coated with αCD3 antibody (17A2, 10 μg/ml) at 4 °C, overnight, at the density 1 × 10^4^ cells/100 μl/well. Next, they were loaded with OVA_323–339_ peptide (2 μg/ml; AnaSpec, Fremont, CA, USA) for 45 min at 37 °C and washed 3 times to remove unbound peptide. Naïve, CD4^+^ OT-II cells (CD45.1/2^+^) were purified using a CD4^+^T cell negative depletion kit (Invitrogen). Cells were further labeled with 1 μM CFSE (Molecular Probes, Eugene, OR, USA) for 10 min at 37 °C and washed twice. They were added to peptide-loaded antigen-presenting cells at a density of 1 × 10^5^ cells/well (antigen-presenting cell/OT-II ratio 1:10). Treg cells (CD45.2^+^) were sorted as CD4^+^CD25^+^ cells from spleens of miR-181a/b-1^+/−^ and miR-181a/b-1^−/−^ mice (purity always >98%), and their graded concentrations were added to antigen-presenting cell/OT-II cocultures in the presence of murine IL-2 (100 U/ml, PeproTech). Assay was analyzed after 48–60 h by flow cytometry.

### In vivo suppression assay

To analyze the function of Treg cells in vivo, sorted populations of naïve CD45.1^+^ (1 × 10^5^ to 4 × 10^5^ cells) together with CD45.2^+^ miR-181a/b-1^+/−^ or miR-181a/b-1^−/−^ Treg cells were injected intravenously into *Rag1*^−/−^ recipients and analyzed after 14 days.

### TCR sequencing

Total RNA was isolated using the RNeasy Mini Kit (Qiagen, Hilden, Germany) from 1 × 10^5^ sorted thymic CD3^+^CD4^+^CD25^+^ miR-181a/b-1^+/−^ or miR-181a/b-1^−/−^ Treg cells and 4 × 10^5^ sorted splenic CD3^+^CD4^+^CD25^+^ miR-181a/b-1^+/−^ or miR-181a/b-1^−/−^ Treg cells. Purity was above 98% as determined by reanalysis. Four independent sorts were performed (pooled 4–5 mice/genotype), and so were 2 independent sequencing experiments. cDNA templates were synthesized using SuperScript II reverse transcriptase (Invitrogen) according to the manufacturer’s recommendation. To generate template libraries of rearranged TCR CDR3 regions from Treg-cell cDNA for the Genome Sequencer FLX system (454 sequencing; Roche, Basel, Switzerland), we used primers spanning the variable region between constant Cα and V elements of the Vα8 family (comprising TRAV12-1*01, TRAV12-1*03, TRAV12-1*04, TRAV12-1*05, TRAV12D-2*01, TRAV12D-2*02, TRAV12D-2*03, TRAV12D-2*04, TRAV12D-2*05, TRAV12D-3*01, TRAV12D-3*02, and TRAV12D-3*03) [50]. Forward and reverse primers contained at their 5′ ends the universal adapter sequences and a multiplex identifier (MID), respectively. Amplicons were purified by agarose gel electrophoresis and QIAquick Gel Extraction Kit (Qiagen) and quantified by Quant-iT dsDNA HS Assay Kit (Invitrogen). Single PCR amplicon molecules were immobilized onto DNA capture beads within an oil–water emulsion to enable clonal amplification in a second PCR process with universal primers. The emulsion was then disrupted, and isolated beads were loaded onto PicoTiterPlates. Sequencing reactions were performed by ultradeep 454 pyrosequencing on the Genome Sequencer FLX system (Roche). Productive rearrangements and CDR3α regions were defined by comparing nucleotide sequences to the reference sequences from IMGT, the international ImMunoGeneTics information system (http://www.imgt.org) [51]. Rearrangements were analyzed and CDR3α regions were defined using IMGT/HighV-QUEST [52].

### Confocal microscopy

CD4^+^ T cells were purified from spleens and pLN of miR-181a/b-1^+/−^ and miR-181a/b-1^−/−^ mice using CD4^+^ T-cell negative isolation kit (Dynal, Invitrogen). Next, cells were plated on glass coverslips (Assistent, 0.13–0.16 mm, Thermo Fisher Scientific) coated with poly-L-lysine (Sigma Aldrich) or Histogrip (Invitrogen) for 2 h at 37 °C. In some experiments, glass coverslips were additionally coated with αCD3 antibody (10 μg/ml, 17A2) and αCD28 antibody (10 μg/ml, 37.51). After 2 h, cells were fixed in 3% (v/v) electron-microscopy–grade PFA (Electron Microscopy Sciences). CTLA-4, Foxp3, LAMP2, EEA1, GM130, and Rab11 were stained using indirect immunofluorescence. The following primary antibodies were used: purified α-mouse CTLA-4 (UC10-4F10-11, BD Biosciences) labeled with DyLight650 antibody labeling kit (Pierce, Thermo Fisher Scientific) according to the manufacturer’s protocol, AlexaFluor488-labeled rat-α-mouse Foxp3 (MF23, BD Biosciences), purified rat-α-mouse LAMP2 (Hybridoma Bank), α-mouse EEA-1 (14/EEA1, BD Biosciences), α-mouse GM130 (35/GM130, BD Biosciences), and α-mouse Rab11 (D4F5, Cell Signaling, Danvers, MA). The following secondary antibodies were used, all conjugated to AlexaFluor488 (all Molecular Probes): mouse-α-rat, rat-α-mouse, and goat-α-rabbit. Nuclear staining was performed using DAPI (Molecular Probes). Coverslips were mounted on glass slides using aqueous mounting medium (DakoCytomation, Glostrup, Denmark). Samples were analyzed by confocal fluorescent microscopy using a Leica SP5 inverted microscope (Leica Microsystems, Wetzlar, Germany). During imaging, a single focal plane was monitored in x-y-z scanning mode using 63×/1.4–0.6 NA oil HCX PL APO lambda blue DIC oil objective, UV laser (405 nm), argon laser (488 nm), diode-pumped solid-state (DPSS) laser (561 nm), and helium–neon (HeNe) laser (633 nm) at a scanner frequency of 400 Hz, line averaging 4. In order to avoid fluorescence overlap, sequential scans were performed. Images were analyzed using LAS AF Lite (Leica Microsystems) and ImageJ software. Quantification of fluorescence intensity and image analysis was performed using ImageJ software.

### Inhibition of lysosomal degradation in Treg cells

CD4^+^CD25^+^ Treg cells were enriched from spleens and LNs of miR-181a/b-1^+/−^ or miR-181a/b-1^−/−^ mice using a MACS isolation kit (Miltenyi Biotec, Bergisch Gladbach, Germany). Cells were incubated in the absence or presence of 50 nM bafilomycin (InvivoGen, San Diego, CA, USA) and collected after 30, 60, 120, and 180 min. Samples were stained with αCD4, αCD25, and αhCD2 (Foxp3) antibodies and intracellularly for CTLA-4. For intracellular stainings, the Foxp3/transcription factor staining buffer set (eBioscience) was used according to the manufacturer’s protocol. Samples were acquired on LSRII (BD Biosciences). Data were analyzed with FlowJo software, v.9.4.9 (Tree Star). For analysis, dead cells and debris were excluded via staining with Zombie Aqua reagent (BioLegend) prior to fixation and permabilization of cells.

### Microarrays

RNA isolation, cDNA preparation, and DNA microarray analysis of gene expression were performed at the Microarray Genechip Facility of the University of Tübingen (MFT Services). Fluorescent images of hybridized microarrays (MOE-430 version 2.0; Affymetrix, Santa Clara, CA, USA) were obtained using an Affymetrix Genechip Scanner. Microarray data were analyzed using BioConductor Suite 2.1 software. All samples were repeated two times with individually sorted cells and averaged.

### Small RNAseq

Treg cells (1 × 10^5^) sorted from 3 pooled WT and miR-181a/b-1^−/−^ thymi were stored in RNAprotect Cell Reagent (Qiagen). Small RNAseq was performed by Admera Health (South Plainfield, NJ, USA) using the SMARTer smRNA-Seq Kit (Takara, Kusatsu, Japan). Adapters were trimmed with Flexbar 3.4, and rRNA was removed using Bowtie 2. The remaining reads were aligned using STAR aligner and counted using HTSeq. Differential expression analysis was performed in R using the DESeq2 package. Three biological replicates per genotype were analyzed.

### Luciferase assay

Part of the CTLA-4 coding sequence (CTLA-4^WT^, 294 bp) and a version devoid of the putative miR-181a binding site (CTLA-4^del^, 271 bp) were synthesized by GeneArt (Regensburg, Germany) and cloned into PsiCheck2.0 Vector (Promega, Madison, WI, USA). 3T3 cells overexpressing murine miR-181a or ctrl cells were established by retroviral transduction and sorted to 100% purity (GFP reporter). Viral particles were produced in HEK293T cells by co-transfection with pCLEco (coexpressing MLV gag, pol, and env) and the plasmids MDH1-PGK-GFP_2.0 (Addgene plasmid #11375) or MDH1-miR-181a-1-PGK-GFP (Addgene plasmid #11376), which were gifts from Chang-Zheng Chen [53]. 3T3 cells were cultured in complete DMEM (Life Technologies, Carlsbad, CA, USA) (10% FCS, 100 U PenStrep, 1 mM Na-pyruvate, 25 μg/mL Geneticin [Life Technologies]). 250,000 cells were electroporated with 0.1 μg of PsiCheck2.0 (250 V, 950 μF, Biorad Gene Pulser II) and cultured for 24 h on 6-well plates. Dual-Luciferase Reporter (DLR) assays (Promega) were conducted according to the manufacturer’s instructions. Luciferase activity was measured using Lumat LB 9507 machine (Berthold).

### qRT-PCR

RNA was prepared using the miRNeasy Kit according to the manufacturer’s instructions (Qiagen). RT reaction was performed using TaqMan MicroRNA Reverse Transcription Kit (Applied Biosystems, Thermo Fisher Scientific, Waltham, MA, USA) and miRNA-specific primers according to the manufacturer’s protocol. Quantitative RT-PCR analysis of miRNA expression was carried out using the following Taqman probes: hsa-miR-181a, TM: 000480; mmu-miR-15b, TM: 000390; mmu-miR-150-5p, TM: 000473; mmu-miR-342, TM: 002260; mmu-let-7g-5p, TM: 002282 (Applied Biosystems). Fold differences were calculated using the ΔC_t_ method normalized to snoRNA412 as housekeeping miRNA gene (Applied Biosystems, TM: 001243).

### Statistical analyses

All analyses were performed using GraphPad Prism software. Data are represented as mean ± SD. Statistical analyses of significance were performed using unpaired or paired Student’s *t* test, multiple *t* test, or two-way ANOVA with Bonferroni post *t* test or Sidak’s multiple comparison test.

## Supporting information

S1 Fig Early development of Treg cells in the absence of miR-181a/b-1. (A) Competitive BM chimeras. BM cells from miR-181a/b-1^+/−^ (het) or miR-181a/b-1^−/−^ (KO) (both CD45.2) were mixed in a 1:1 ratio with competitor WT BM cells (CD45.1/2) and injected into lethally irradiated WT recipients (CD45.1). Chimeras were analyzed 12 weeks later for the generation of CD4^−^CD8α^−^ (DN), CD4^+^CD8α^+^ (DP), and CD4^+^CD8α^−^ (CD4^+^SP) cells in the thymus. Plots are representative of 2 independent experiments with *n* = 8 for each genotype. Graph shows ratio of cells within test versus competitor populations. Each data point represents an individual mouse. (B) FACS analysis of thymi from InduRag1 mice sufficient and deficient for miR-181a/b-1, 7 days after induction of Rag1 expression. (C) Frequencies of tTreg cells (TCRβ^+^CD4^+^CD25^+^Foxp3^+^), their precursors TCRβ^+^CD4^+^CD25^−^Foxp3^+^ (Ia), and TCRβ^+^CD4^+^CD25^+^Foxp3^−^ (Ib) on day 7 after initial induction of Rag1 expression in InduRag1 mice sufficient and deficient for miR-181a/b-1. (D) FACS analysis of CD4 T-cell selection, 7 days after Rag1 induction in InduRag1 mice. Depicted data are from 2 independent experiments, with *n* = 1–4 for each genotype and time point analyzed. Numerical values are available in S1 Data. BM, bone marrow; CD, cluster of differentiation; DN, double negative; DP, double positive; FACS, fluorescence-activated cell scan; Foxp3, forkhead box protein P3; InduRag1, inducible recombination-activating gene 1; KO, knockout; miR-181, microRNA-181; prec, precursor; *Rag1*, recombination-activating gene 1; SP, single positive; Treg cell, regulatory T cell; tTreg cell, thymic Treg cell; WT, wild type.
(JPG)

S2 Fig miR-181a/b-1 deficiency impairs generation of Treg cells in the thymus and does not influence their peripheral induction from naïve T cells. (A) Plots show gating strategy to discriminate newly generated tTreg cells (Foxp3^hCD2+^ cells positive for *Rag1*^GFP^) and the population consisting of peripheral immigrants and thymus-residing mature Treg cells (Foxp3^hCD2+^GFP^−^ Treg cells). Representative plots of 5 independent experiments are shown. Right panel, quantification; each data point represents one mouse. (B) Frequencies of RTEs (*Rag1*^GFP+^CD4^+^CD25^−^) in spleens of miR-181a/b-1^+/−^ and miR-181a/b-1^−/−^ mice, left panel. Frequencies of Treg-cell RTEs (*Rag1*^GFP+^CD4^+^Foxp3^hCD2+^) in spleens of miR-181a/b-1^+/−^ and miR-181a/b-1^−/−^ mice, right panel. (C) De novo induction of Treg cells is not enhanced in the absence of miR-181a/b-1. RTEs (*Rag1*^GFP+^CD4^+^CD25^−^) were sorted from spleens of *Rag1*^GFP/WT^ × miR-181a/b-1–sufficient and deficient mice (CD45.1^+^/CD45.2^+^ or CD45.2^+^) and injected into lymphopenic *Il7ra*^−/−^ recipients (CD45.1). Generation of Treg cells within donor cells was analyzed after 28 days. Plots are representative of 2 independent experiments, *n* = 3–4. Graphs show frequencies of CD25^+^Foxp3^+^ cells generated within donor TCRβ^+^CD4^+^ cells in spleen, pLNs, and mLNs. Statistical analysis was performed using unpaired Student’s *t* test. Numerical values are available in S1 Data. CD, cluster of differentiation; Foxp3, forkhead box protein P3; GFP, green fluorescent protein; hCD2, human CD2; *Il7r*, interleukin-7 receptor alpha; miR-181, microRNA-181; mLN, mesenteric lymph node; pLN, peripheral lymph node; *Rag1*, recombination activating gene 1; RTE, recent thymic emigrant; TCR, T-cell receptor; Treg cell, regulatory T cell; tTreg cell, thymic Treg cell.
(JPG)

S3 Fig Peripheral expansion of miR-181a/b-1–deficient Treg cells compensates for their impaired development. (A) TCR repertoire of miR-181a/b-1–deficient tTreg (upper graph) and splenic Treg (lower graph) cells. Vα8–Cα amplicons were amplified from cDNA of Treg cells before high-throughput sequencing. Frequencies of individual Vα–Cα sequences detected in miR-181a/b-1^+/−^ (black) and miR-181a/b-1^−/−^ (red) Treg cells. The copy number of Vα–Cα sequences is displayed against the number of individual nucleotide sequences. Data are derived from 2 independent experiments with samples sorted from *n* = 4–6 mice (pool). Numerical values are available in S1 Data. cDNA, complementary DNA; miR-181, microRNA-181; TCR, T-cell receptor; Treg cell, regulatory T cell; tTreg cell, thymic Treg cell.
(JPG)

S4 Fig Flow-cytometry analysis of miR-181a/b-1–deficient Treg cells. Selected surface and intracellular proteins expressed by tTreg (A), splenic Treg (B), and LN-resident Treg (C) cells. Representative histograms and plots from 2 independent experiments (*n* = 6–9 for each genotype) are depicted. Numbers indicate average MFI or frequencies of positive cells, ±SD. Numerical values are available in S1 Data. LN, lymph node; MFI, mean fluorescence intensity; miR-181, microRNA-181; Treg cell, regulatory T cell; tTreg cell, thymic Treg cell.
(JPG)

S5 Fig No evidence for post-transcriptional regulation of CTLA-4 by miR-181a/b-1 or miRNAs down-regulated in miR-181a/b-1–deficient Treg cells. (A) Predicted base-pairing of miR-181a with the target sequence in the cds of CTLA-4. The seed sequence in the miRNA and the complementary sequence in the cds are displayed in bold letters. Number indicates the position within the CTLA-4 cds. (B) Relative luciferase intensities of CTLA-4 coding sequence (CTLA-4^WT^) and cds lacking 23 bp of the predicted miR-181a binding site (CTLA-4^del^) normalized to empty luciferase vector ctrl in 3T3 cells overexpressing miR-181a (miR-181a) or respective ctrls. Bars represent mean of >20 experiments and SD. (C) Small RNAseq volcano plot of differentially regulated miRNAs in miR-181a/b-1^−/−^ compared to WT tTreg cells. (D) qRT-PCR analysis of differentially regulated miRNAs identified in small RNAseq analysis in sorted tTreg cell (left column) and splenic Treg cell populations (right column). Data from 3 independent experiments, with *n* = 2–7 (pool) for each genotype. Expression of each miRNA was normalized to the expression of housekeeping small RNA, snoR412. ΔΔC_T_ values are displayed on the graph. Numerical values are available in S1 Data. cds, coding sequence; CTLA-4, cytotoxic T-lymphocyte–associated protein 4; ctrl, control; miRNA, microRNA; miR-181, microRNA-181; qRT-PCR, quantitative reverse-transcription PCR; RNAseq, RNA sequencing; snoR412, small nucleolar RNA 412; Treg cell, regulatory T cell; tTreg cell, thymic Treg cell; WT, wild type.
(JPG)

S6 Fig miR-181a/b-1–deficient Treg cells are more suppressive in vitro. (A) Production of cytokines by splenic CD8^+^ T cells after stimulation with PMA/ionomycin. Graphs represent quantification of the data from 2 independent experiments, *n* = 4–5 for each genotype. (B) In vitro suppression assay. Splenic antigen-presenting cells were loaded with OVA_323–339_ peptide and cocultured with OT-II cells in the presence of graded numbers of sorted Treg cells from spleens of miR-181a/b-1^+/−^ and miR-181a/b-1^−/−^ mice. Graph shows percent of suppression calculated as follows: The number of CFSE^low^ OT-II cells (dividing) in the absence of Treg cells (ctrl sample) was set as 100%. Further, numbers of CFSE^low^ OT-II cells that survived in the presence of Treg cells were transformed to frequencies according to ctrl sample, and this number was subtracted from 100%, which gave the percent of suppression exhibited by a given number of Treg cells. Data are representative of 4 independent experiments, with *n* = 7–8 for Treg cell donor mice. Numerical values are available in S1 Data. CFSE, carboxyfluorescein succinimidyl ester; ctrl, control; miR-181, microRNA-181; OT-II, ovalbumin-specific MHC class II-restricted alpha beta TCR; OVA, chicken ovalbumin; PMA, phorbol 12-myristate 13-acetate; Treg cell, regulatory T cell.
(JPG)

S1 Data Numerical values for graphical representations of data shown in all figures. (XLSX)

## Abbreviations

APC: allophycocyanin
BM: bone marrow
CD4: cluster of differentiation 4
CHX: cycloheximide
Cre: Cre recombinase
CTLA-4: cytotoxic T-lymphocyte–associated protein 4
DLR: Dual-Luciferase Reporter
DP: CD4^+^CD8^+^ double positive
DPSS: diode-pumped solid state
*Dusp6*: dual specificity phosphatase 6
EEA1: early endosome antigen 1
ERT2: tamoxifen-inducible estrogen receptor 2
FACS: fluorescence-activated cell scan
FITC: fluorescein isothiocyanate
Foxp3: forkhead box protein P3
GFP: green fluorescent protein
GITR: glucocorticoid-induced tumor necrosis factor receptor-related protein
GM130: *cis*-Golgi matrix protein 130
hCD2: human cluster of differentiation 2
IL: interleukin
*Il7r*: interleukin-7 receptor α gene
InduRag1: inducible recombination-activating gene 1
iNKT: invariant natural killer T
ITAM: immunoreceptor tyrosine-based activation motif
iTreg: induced Treg
KO: knockout
LAMP2: lysosome-associated membrane protein 2
LAVES: Niedersächsisches Landesamt für Verbraucherschutz und Lebensmittelsicherheit
LBD: ligand-binding domain
let-7g: lethal-7g
lin: lineage
LSK: lineage-negative, stem cell antigen-1-positive, Kit-positive
MAIT: Mucosal-Associated Invariant T
MFI: mean fluorescence instensity
MHC: major histocompatibility complex
MID: multiplex identifier
miR-181a/b-1: microRNA family members 181a-1 and 181b-1
miRNA: microRNA
mLN: mesenteric lymph node
*Nr4a1*: Nuclear receptor subfamily 4 group A member 1
Nur77: Nuclear hormone receptor NUR/77 encoded by *Nr4a1*
OT-II: ovalbumin-specific MHC class II-restricted alpha beta TCR
OVA: chicken ovalbumin
PE: phycoerythrin
PerCP-Cy5.5: peridinin chlorophyll protein-Cy5.5
pLN: peripheral lymph node
PMA: phorbol 12-myristate 13-acetate
prec: precursor
*Ptpn*: protein tyrosine phosphatase, nonreceptor type
qRT-PCR: quantitative reverse-transcription PCR
Rab11: Ras-related in brain protein 11
*Rag1*: recombination activating gene 1
RIPmOVA: rat insulin promoter-driven membrane-bound chicken ovalbumin
RNAseq: RNA sequencing
RTE: recent thymic emigrant
scLN: subcutaneous lymph node
snoR412: small nucleolar RNA 412
SP: single positive
Tconv: conventional T cell
TCR: T-cell receptor
TNF: tumor necrosis factor
Treg cell: regulatory T cell
tTreg: thymic Treg
WT: wild type
Zap-70: Zeta-chain–associated tyrosine protein kinase of 70 kD
