# Supplementary figures and images for: miR-181a/b-1 controls thymic selection of Treg cells and tunes their suppressive capacity
Source: PLoS Biol. 2019 Mar 11;17(3):e2006716. doi: 10.1371/journal.pbio.2006716 (PMC6428341; doi:10.1371/journal.pbio.2006716)

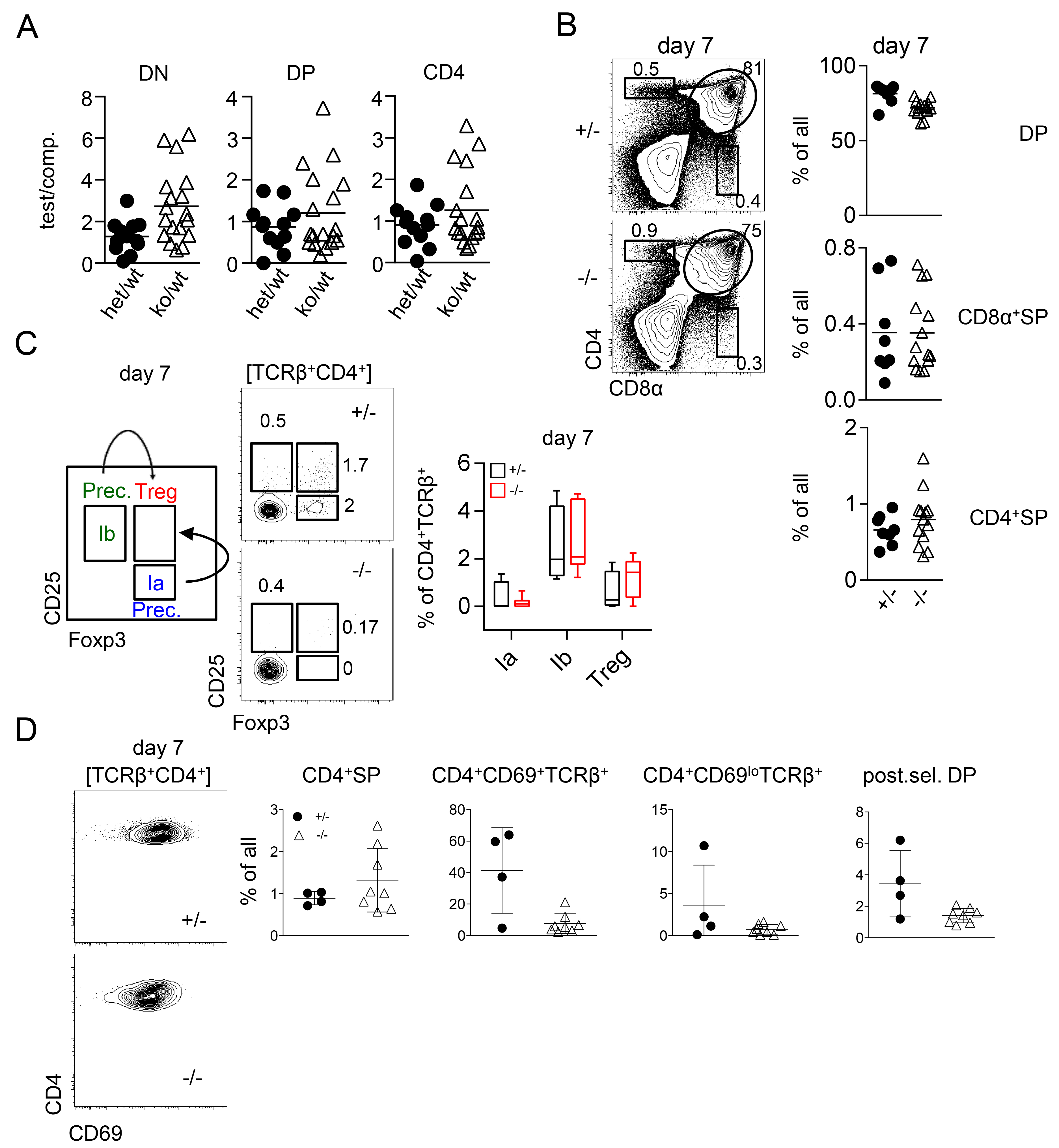

Supplement: S1 Fig — (A) Competitive BM chimeras. BM cells from miR-181a/b-1+/− (het) or miR-181a/b-1−/− (KO) (both CD45.2) were mixed in a 1:1 ratio with competitor WT BM cells (CD45.1/2) and injected into lethally irradiated WT recipients (CD45.1). Chimeras were analyzed 12 weeks later for the generation of CD4−CD8α− (DN), CD4+CD8α+ (DP), and CD4+CD8α− (CD4+SP) cells in the thymus. Plots are representative of 2 independent experiments with n = 8 for each genotype. Graph shows ratio of cells within test versus competitor populations. Each data point represents an individual mouse. (B) FACS analysis of thymi from InduRag1 mice sufficient and deficient for miR-181a/b-1, 7 days after induction of Rag1 expression. (C) Frequencies of tTreg cells (TCRβ+CD4+CD25+Foxp3+), their precursors TCRβ+CD4+CD25−Foxp3+ (Ia), and TCRβ+CD4+CD25+Foxp3− (Ib) on day 7 after initial induction of Rag1 expression in InduRag1 mice sufficient and deficient for miR-181a/b-1. (D) FACS analysis of CD4 T-cell selection, 7 days after Rag1 induction in InduRag1 mice. Depicted data are from 2 independent experiments, with n = 1–4 for each genotype and time point analyzed. Numerical values are available in S1 Data. BM, bone marrow; CD, cluster of differentiation; DN, double negative; DP, double positive; FACS, fluorescence-activated cell scan; Foxp3, forkhead box protein P3; InduRag1, inducible recombination-activating gene 1; KO, knockout; miR-181, microRNA-181; prec, precursor; Rag1, recombination-activating gene 1; SP, single positive; Treg cell, regulatory T cell; tTreg cell, thymic Treg cell; WT, wild type. (JPG) [file pbio.2006716.s001.jpg]

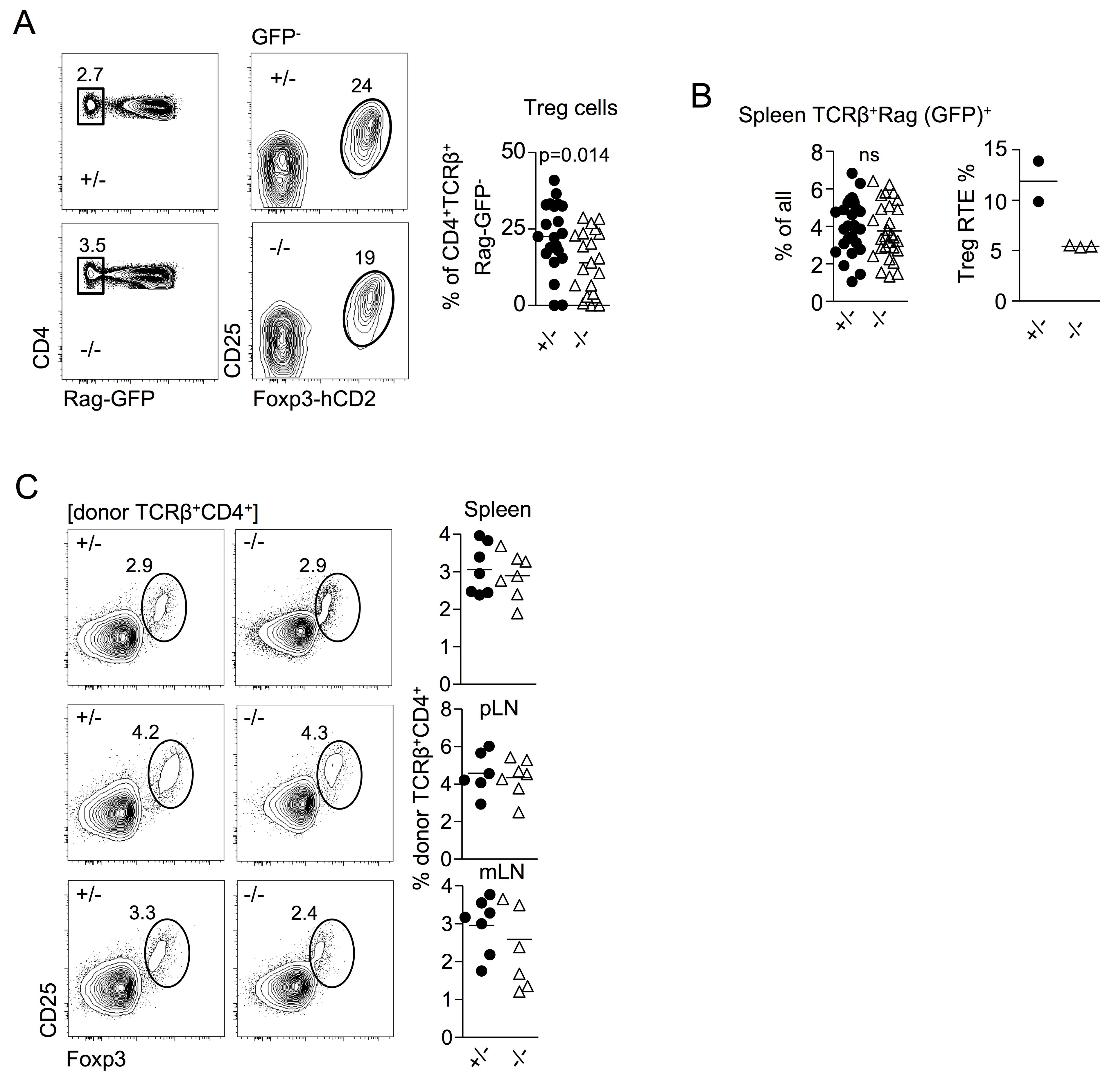

Supplement: S2 Fig — (A) Plots show gating strategy to discriminate newly generated tTreg cells (Foxp3hCD2+ cells positive for Rag1GFP) and the population consisting of peripheral immigrants and thymus-residing mature Treg cells (Foxp3hCD2+GFP− Treg cells). Representative plots of 5 independent experiments are shown. Right panel, quantification; each data point represents one mouse. (B) Frequencies of RTEs (Rag1GFP+CD4+CD25−) in spleens of miR-181a/b-1+/− and miR-181a/b-1−/− mice, left panel. Frequencies of Treg-cell RTEs (Rag1GFP+CD4+Foxp3hCD2+) in spleens of miR-181a/b-1+/− and miR-181a/b-1−/− mice, right panel. (C) De novo induction of Treg cells is not enhanced in the absence of miR-181a/b-1. RTEs (Rag1GFP+CD4+CD25−) were sorted from spleens of Rag1GFP/WT × miR-181a/b-1–sufficient and deficient mice (CD45.1+/CD45.2+ or CD45.2+) and injected into lymphopenic Il7ra−/− recipients (CD45.1). Generation of Treg cells within donor cells was analyzed after 28 days. Plots are representative of 2 independent experiments, n = 3–4. Graphs show frequencies of CD25+Foxp3+ cells generated within donor TCRβ+CD4+ cells in spleen, pLNs, and mLNs. Statistical analysis was performed using unpaired Student’s t test. Numerical values are available in S1 Data. CD, cluster of differentiation; Foxp3, forkhead box protein P3; GFP, green fluorescent protein; hCD2, human CD2; Il7r, interleukin-7 receptor alpha; miR-181, microRNA-181; mLN, mesenteric lymph node; pLN, peripheral lymph node; Rag1, recombination activating gene 1; RTE, recent thymic emigrant; TCR, T-cell receptor; Treg cell, regulatory T cell; tTreg cell, thymic Treg cell. (JPG) [file pbio.2006716.s002.jpg]

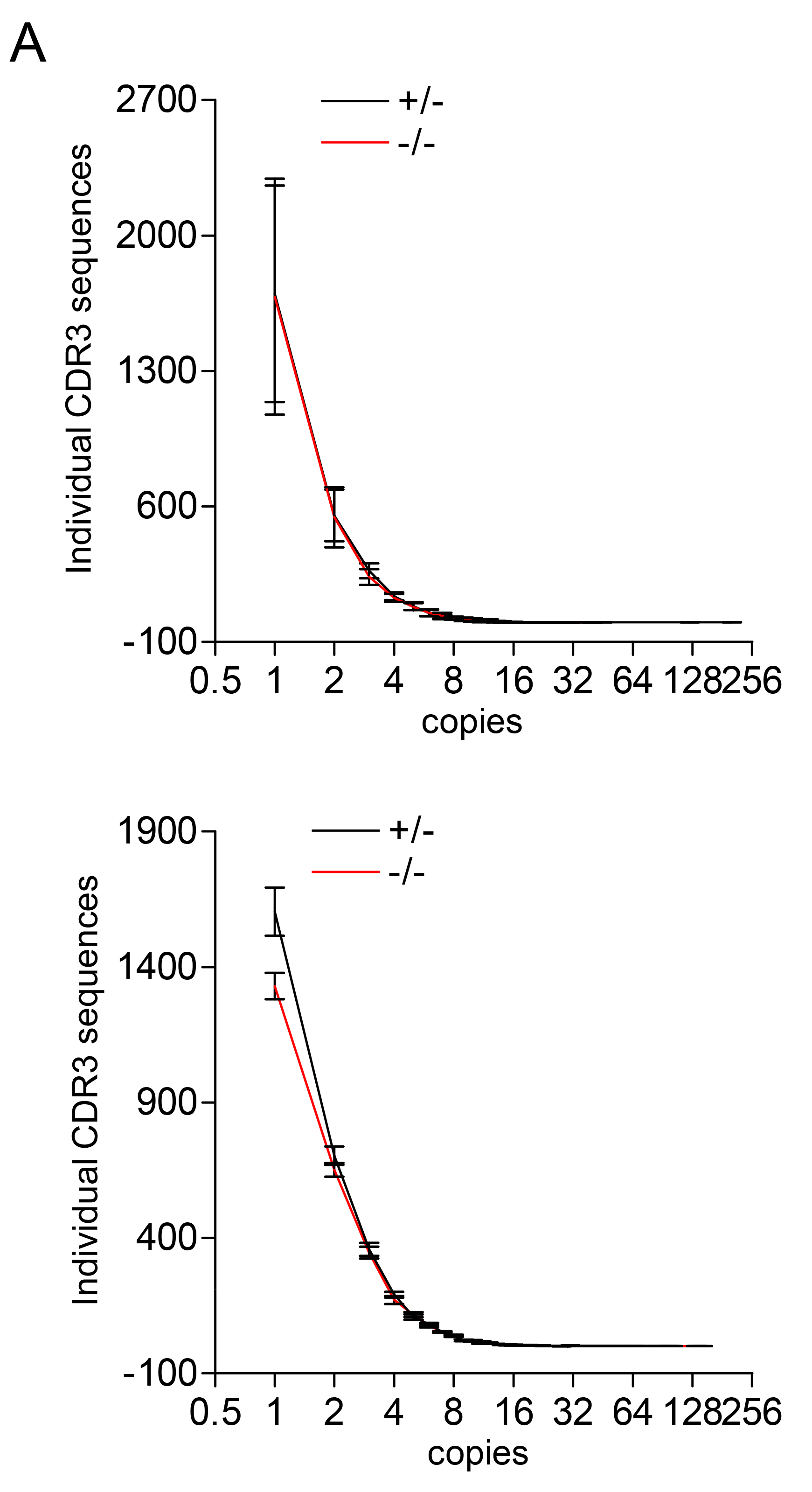

Supplement: S3 Fig — (A) TCR repertoire of miR-181a/b-1–deficient tTreg (upper graph) and splenic Treg (lower graph) cells. Vα8–Cα amplicons were amplified from cDNA of Treg cells before high-throughput sequencing. Frequencies of individual Vα–Cα sequences detected in miR-181a/b-1+/− (black) and miR-181a/b-1−/− (red) Treg cells. The copy number of Vα–Cα sequences is displayed against the number of individual nucleotide sequences. Data are derived from 2 independent experiments with samples sorted from n = 4–6 mice (pool). Numerical values are available in S1 Data. cDNA, complementary DNA; miR-181, microRNA-181; TCR, T-cell receptor; Treg cell, regulatory T cell; tTreg cell, thymic Treg cell. (JPG) [file pbio.2006716.s003.jpg]

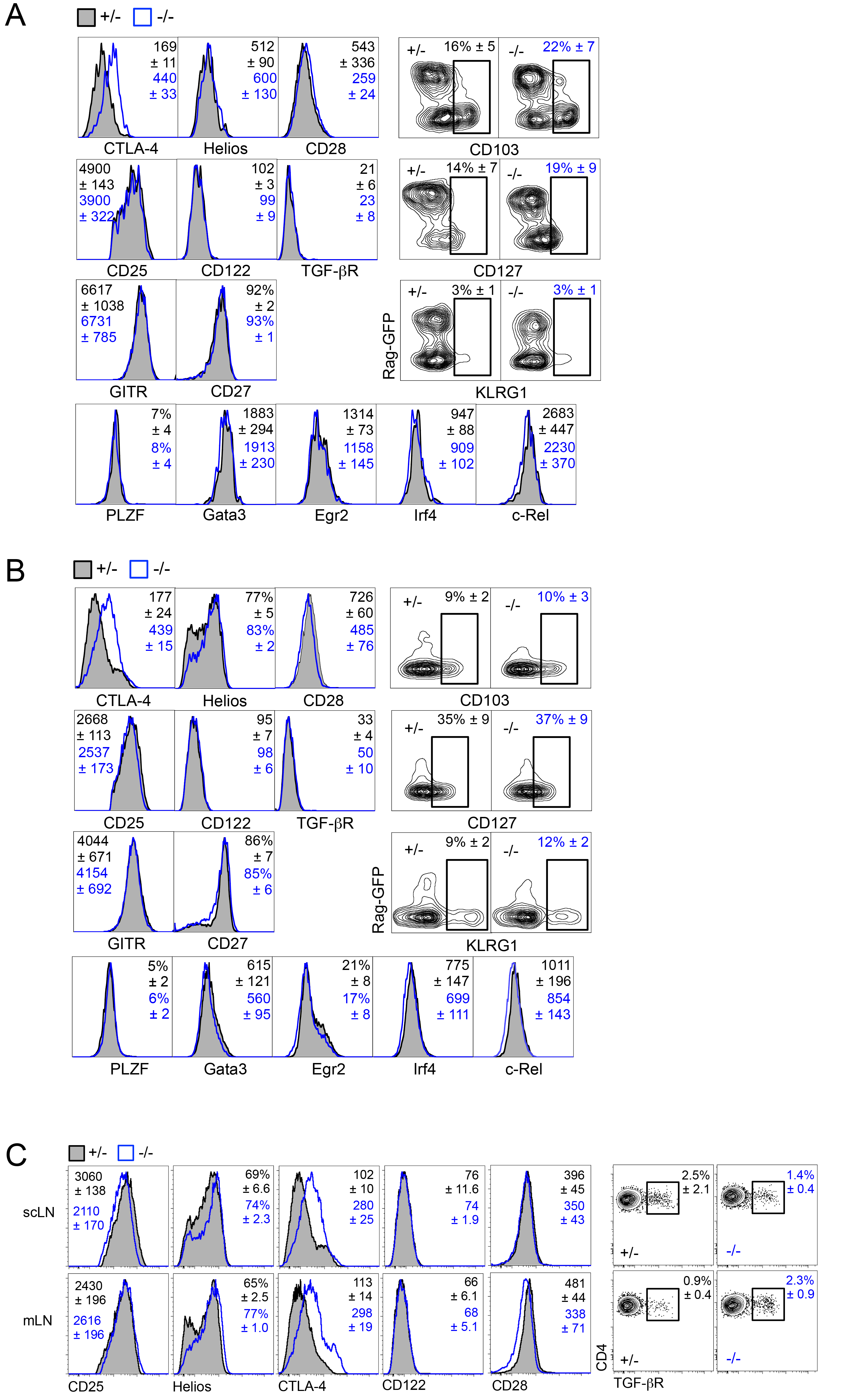

Supplement: S4 Fig — Selected surface and intracellular proteins expressed by tTreg (A), splenic Treg (B), and LN-resident Treg (C) cells. Representative histograms and plots from 2 independent experiments (n = 6–9 for each genotype) are depicted. Numbers indicate average MFI or frequencies of positive cells, ±SD. Numerical values are available in S1 Data. LN, lymph node; MFI, mean fluorescence intensity; miR-181, microRNA-181; Treg cell, regulatory T cell; tTreg cell, thymic Treg cell. (JPG) [file pbio.2006716.s004.jpg]

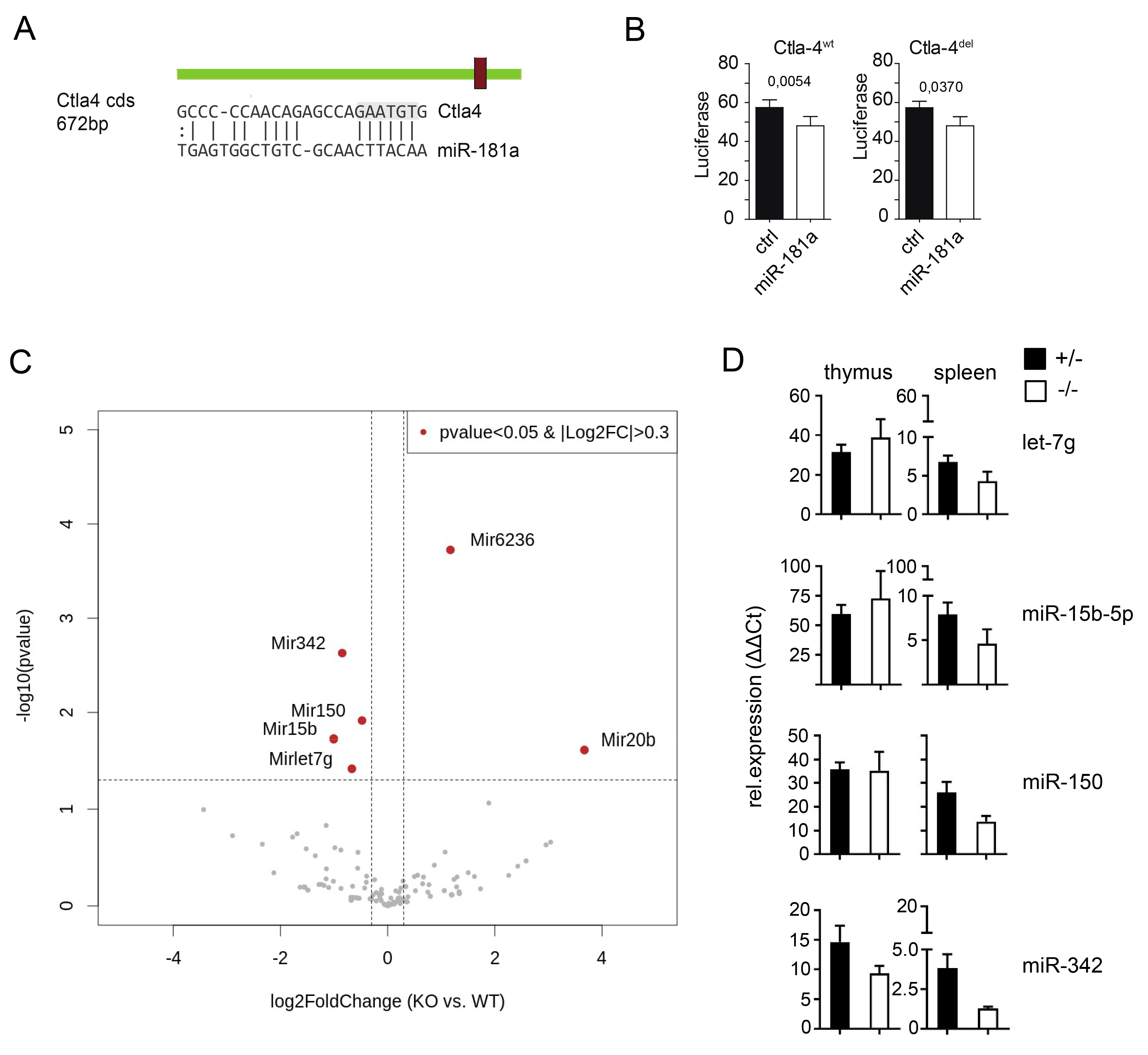

Supplement: S5 Fig — (A) Predicted base-pairing of miR-181a with the target sequence in the cds of CTLA-4. The seed sequence in the miRNA and the complementary sequence in the cds are displayed in bold letters. Number indicates the position within the CTLA-4 cds. (B) Relative luciferase intensities of CTLA-4 coding sequence (CTLA-4WT) and cds lacking 23 bp of the predicted miR-181a binding site (CTLA-4del) normalized to empty luciferase vector ctrl in 3T3 cells overexpressing miR-181a (miR-181a) or respective ctrls. Bars represent mean of >20 experiments and SD. (C) Small RNAseq volcano plot of differentially regulated miRNAs in miR-181a/b-1−/− compared to WT tTreg cells. (D) qRT-PCR analysis of differentially regulated miRNAs identified in small RNAseq analysis in sorted tTreg cell (left column) and splenic Treg cell populations (right column). Data from 3 independent experiments, with n = 2–7 (pool) for each genotype. Expression of each miRNA was normalized to the expression of housekeeping small RNA, snoR412. ΔΔCT values are displayed on the graph. Numerical values are available in S1 Data. cds, coding sequence; CTLA-4, cytotoxic T-lymphocyte–associated protein 4; ctrl, control; miRNA, microRNA; miR-181, microRNA-181; qRT-PCR, quantitative reverse-transcription PCR; RNAseq, RNA sequencing; snoR412, small nucleolar RNA 412; Treg cell, regulatory T cell; tTreg cell, thymic Treg cell; WT, wild type. (JPG) [file pbio.2006716.s005.jpg]

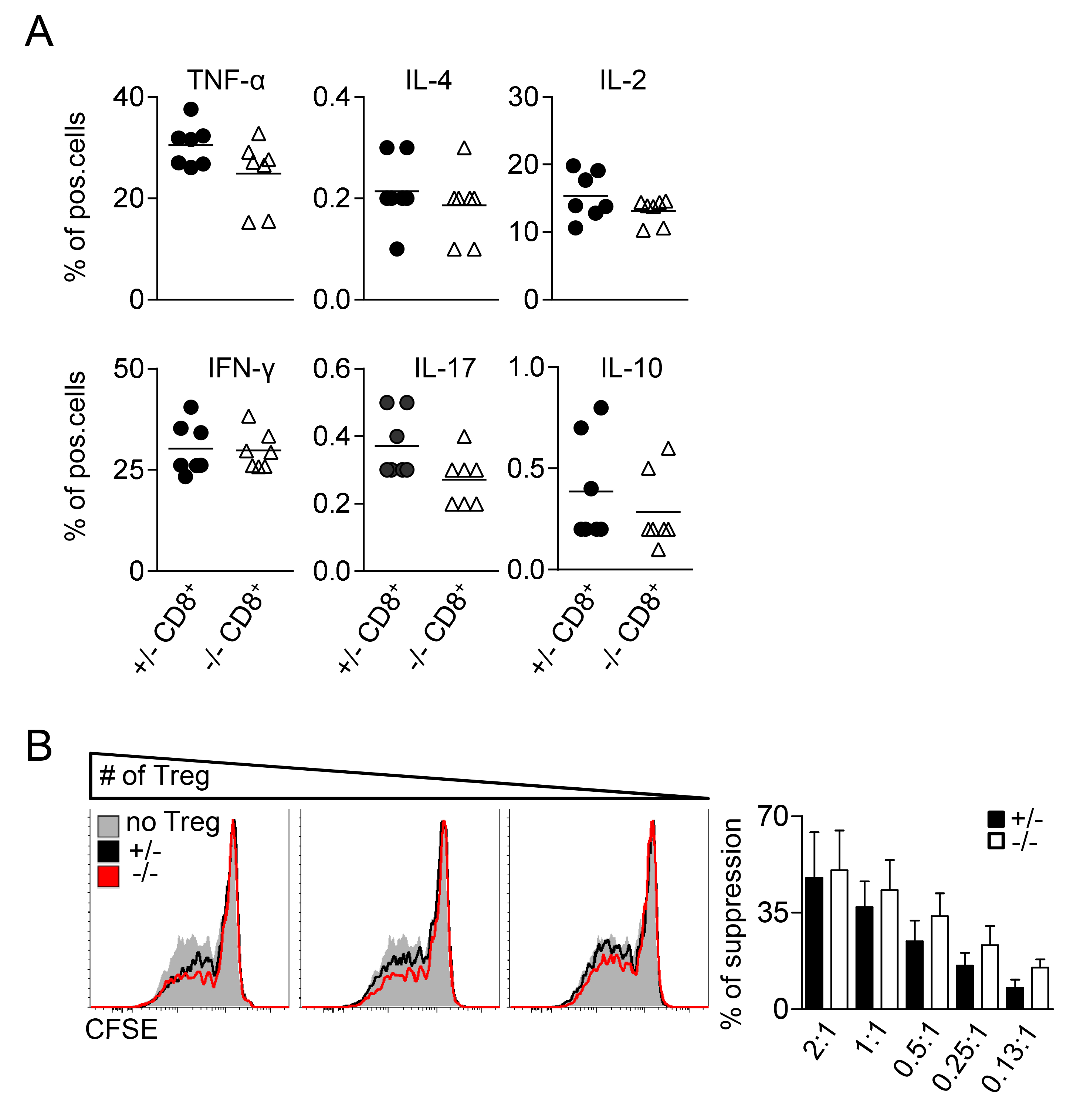

Supplement: S6 Fig — (A) Production of cytokines by splenic CD8+ T cells after stimulation with PMA/ionomycin. Graphs represent quantification of the data from 2 independent experiments, n = 4–5 for each genotype. (B) In vitro suppression assay. Splenic antigen-presenting cells were loaded with OVA323–339 peptide and cocultured with OT-II cells in the presence of graded numbers of sorted Treg cells from spleens of miR-181a/b-1+/− and miR-181a/b-1−/− mice. Graph shows percent of suppression calculated as follows: The number of CFSElow OT-II cells (dividing) in the absence of Treg cells (ctrl sample) was set as 100%. Further, numbers of CFSElow OT-II cells that survived in the presence of Treg cells were transformed to frequencies according to ctrl sample, and this number was subtracted from 100%, which gave the percent of suppression exhibited by a given number of Treg cells. Data are representative of 4 independent experiments, with n = 7–8 for Treg cell donor mice. Numerical values are available in S1 Data. CFSE, carboxyfluorescein succinimidyl ester; ctrl, control; miR-181, microRNA-181; OT-II, ovalbumin-specific MHC class II-restricted alpha beta TCR; OVA, chicken ovalbumin; PMA, phorbol 12-myristate 13-acetate; Treg cell, regulatory T cell. (JPG) [file pbio.2006716.s006.jpg]
